# Improved myelin water imaging using B _1_^+^ correction and data-driven global feature extraction: Application on people with MS

**DOI:** 10.1162/imag_a_00254

**Published:** 2024-07-31

**Authors:** Sharon Zlotzover, Noam Omer, Dvir Radunsky, Neta Stern, Tamar Blumenfeld-Katzir, Dominique Ben-Ami Reichman, Shai Shrot, Chen Hoffmann, Noam Ben-Eliezer

**Affiliations:** Department of Biomedical Engineering, Tel Aviv University, Tel Aviv, Israel; Department of Diagnostic Imaging, Sheba Medical Center, Ramat-Gan, Israel; Sackler School of Medicine, Tel-Aviv University, Tel-Aviv, Israel; Sagol School of Neuroscience, Tel Aviv University, Tel Aviv, Israel; Center for Advanced Imaging Innovation and Research, New York University Langone Medical Center, New York, NY, United States

**Keywords:** myelin water imaging, feature extraction, quantitative MRI, spectral analysis

## Abstract

The predominant technique for quantifying myelin content in the white matter is multicompartment analysis of MRI’s T_2_relaxation times (**mcT_2_**analysis). The process of resolving the T_2_spectrum at each voxel, however, is highly ill-posed and remarkably susceptible to noise and to inhomogeneities of the transmit field (**B_1_^+^**). To address these challenges, we employ a preprocessing stage wherein a spatially global data-driven analysis of the tissue is performed to identify a set of mcT_2_configurations (**motifs**) that best describe the tissue under investigation, followed by using this basis set to analyze the signal in each voxel. This procedure is complemented by a new algorithm for correcting B_1_^+^inhomogeneities, lending the overall fitting process with improved robustness and reproducibility. Successful validations are presented using numerical and physical phantoms vs. ground truth, showcasing superior fitting accuracy and precision compared with conventional (non-data-driven) fitting.*In vivo*application of the technique is presented on 26 healthy subjects and 29 people living with multiple sclerosis (**MS**), revealing substantial reduction in myelin content within normal-appearing white matter regions of people with MS (i.e., outside obvious lesions), and confirming the potential of data-driven myelin values as a radiological biomarker for MS.

## 1 Introduction

Myelin holds a pivotal role in the central nervous system (**CNS**), serving as a protective insulating sheath that envelops nerve fibers. This offers a crucial structural reinforcement and ensures the efficient transmission of electrical signals (Stadelmann et al., 2019). Consequently, the quantification of myelination levels is an invaluable biomarker with a wide range of applications. These include the study of heathy brain development, as well as investigation of neurodegenerative disorders such as Alzheimer’s disease (Dean et al., 2017), Parkinson’s disease (Dean et al., 2016), and multiple sclerosis (**MS**) (Laule et al., 2004;Laule & Moore, 2018;Loma & Heyman, 2011), where myelin mapping can enhance the understanding of these conditions, and facilitate their diagnosis, treatment, and disease progression.

Considering its significance, various MRI techniques have been developed for myelin water imaging (**MWI**), a reliable proxy for myelin content. The most prevalent among these methods are relaxometry-based techniques that rely on the difference between the rapid relaxation rate of water trapped between myelin sheaths and the relaxation rates of the intra-/extracellular water pools. Prominent techniques within this category include gradient and spin echo (**GRASE**) (Prasloski, Rauscher, et al., 2012), multicomponent driven equilibrium single-pulse observation of T_1_and T_2_(**mcDESPOT**) (Deoni et al., 2013;Zhang et al., 2015), T_2_magnetization prepared imaging (Nguyen et al., 2012;Oh et al., 2006), and magnetic resonance fingerprinting (Y. Chen et al., 2019;Nagtegaal, Koken, Amthor, & Doneva, 2020). To fully utilize the differences in T_2_relaxation times and avoid biases caused by field inhomogeneities, approaches based on spin echo protocols are favorable. Signal processing then involves multicomponent T_2_(**mcT_2_**) analysis (Does, 2018;Whittall et al., 1997), where one maps the relative fraction of the fast-relaxing T_2_component (<40 ms) corresponding to water bound between myelin sheaths, and the longer T_2_component (40–200 ms) corresponding to intra-/extracellular water (MacKay & Laule, 2016). The myelin water fraction (**MWF**) serves as an indirect measure of myelin content and is calculated as the ratio of the T_2_spectral content corresponding to myelin water to the total area under the T_2_spectrum.

The most efficient protocol for mcT_2_mapping*in vivo*is 2D (i.e., multislice) multiecho spin echo (**MESE**), offering high-sensitivity T_2_relaxation times at clinically relevant scan times of 5–8 minutes. MESE signals, however, are contaminated by stimulated and indirect echoes (Hennig, 1988), thereby deviating from ideal multiexponential behavior. Various strategies have been proposed to mitigate these effects, e.g., shifting to 3D MESE acquisitions (Dvorak et al., 2020;Meyers et al., 2009;Prasloski, Mädler, et al., 2012), adjusting the refocusing slice width to be significantly larger than the excitation slice profile (Faizy et al., 2016;Kumar et al., 2016;Pell et al., 2006), or using complex crusher gradient schemes (Kolind et al., 2009;Mackay et al., 1994). A more effective solution is the utilization of advanced algorithms such as the extended phase graph (**EPG**) method, which iteratively traces the evolution of multiple spin populations throughout an MESE echo train by incorporating the stimulated echoes into their signal model (Hennig, 1988;Lebel & Wilman, 2010). Another efficient approach is the echo modulation curve (**EMC**) algorithm, which employs Bloch simulations to comprehensively account for all coherence pathways arising during the echo train, thereby faithfully reproducing both stimulated and indirect echoes (Ben-Eliezer et al., 2015,^2016^;McPhee & Wilman, 2017;Radunsky et al., 2021).

Once a model is chosen for resolving the bias due to stimulated echoes, the fundamental approach to multicomponent analysis involves the inversion of the signal acquisition process. This inversion aims to identify the signals originating from distinct cellular water pools and extract the relative signal intensities associated with each of these pools. This inverse problem is inherently ill-posed and poses a significant challenge, even when incorporating various regularization techniques. Specifically, this inversion process exhibits nonuniqueness in the solutions space and a high susceptibility to noise (Graham et al., 1996;Whittall & MacKay, 1989). Consequently, despite its paramount relevance of myelin mapping for clinical applications, the field of MWI still lacks a gold standard which can effectively address these complexities.

In this study, we utilize a new paradigm for mcT_2_fitting, which effectively mitigates issues related to ambiguity to produce reliable MWF maps. The novel aspect of this technique is a preprocessing step which performs statistical analysis of the signals from the entire WM in order to identify a set of multicomponent configurations (termed mcT_2_‘**motifs’**) which best describe the examined tissue. These motifs are then used as basis functions for a regularized non-negative least square (**RNNLS**) mcT_2_fitting of the signal within each voxel (Omer et al., 2022). To further enhance its accuracy, our approach relies on the EMC signal model, which incorporates the exact pulse-sequence scheme and scan parameters to address the existence of stimulated and indirect echoes. This integration ensures the provision of accurate and reproducible T_2_values that remain consistent across scanners and scan settings (Ben-Eliezer et al., 2015,^2016^;Radunsky et al., 2021). Important methodological improvements are introduced in this study including accounting for transmit field ($B_{1}^{+}$) inhomogeneities, the use of entropy-based regularization constraint, and enforcing pseudo-orthogonality among mcT_2_motifs. Validations are presented on a numerical phantom at varying SNRs, and on a physical three-compartment phantom having a unique design which offers ground truth values. Performance of the data-driven approach is also evaluated vis-à-vis conventional RNNLS processing. Clinical applicability of the new approach is demonstrated on healthy subjects and people with MS.

## 2 Theory

### 2.1 Conventional EMC-based mcT _2_ analysis

For readers’ convenience, we provide a concise overview of the conventional, nondata-driven approach to mcT_2_fitting of MESE signals using the EMC signal model and RNNLS fitting. A comprehensive description of this method is also available inOmer et al. (2022).

The MESE signal from each voxel represents a superposition of signals from several subvoxel (cellular) water pools. Due to contamination by stimulated and indirect echoes, attempting to model this signal using a multiexponential decay approach will lead to inaccuracies. To address this issue, we employ the EMC algorithm, which generates realistic T_2_decay curves by accurately simulating the exact pulse-sequence scheme, radiofrequency (**RF**) and gradient pulse shapes, and timing diagram of the 2D MESE protocol, used for image acquisition. The signal from each voxel can then be modeled as:

$$s=\sum_{i=1}^{N_{T_{2}}}w_{i}\cdot d_{i}=Dw,$$ (1)

where$s\in {\mathbb{R}}^{ETL}$is the experimentally acquired signal,$D\in {\mathbb{R}}^{ETL\times N_{T_{2}}}$is a simulated dictionary of single T_2_EMC signals ($d_{i}$),$w\in {\mathbb{R}}^{N_{T_{2}}}$is the relative fraction of each subvoxel water pool, ETL is the echo train length, and$N_{T_{2}}$is the number of cellular components. Extraction ofwis typically done by solving an RNNLS minimization problem of the form:

$$\underset{w}{\text{argmin}}\frac{1}{2}{\left\|Dw-\text{s}\right\|}_{2}^{2}+\lambda_{Tikh}{\left\|w\right\|}_{2}^{2}+\lambda_{L_{1}}{\left\|w\right\|}_{1},\;\;\;suchthatw_{i}\geq 0,$$ (2)

where$\lambda_{Tikh},\lambda_{L_{1}}\geq 0$are Tikhonov and L_1_regularization terms. Tikhonov regularization is added to favor smoother solutions, while the L_1_regularization is added to promote sparse solutions. Due to the large space of possible mcT_2_combinations, more than one solution can match each experimental signal. Adding to that the ambiguity caused by noise, solutions of this system of equations tend to be highly unstable and, in many cases, converge toward a wrong local minimum.

### 2.2 Data-driven EMC-based mcT _2_ analysis

In order to address the inherent ill-posed nature of the inverse problem, we propose the implementation of a data-driven preprocessing stage aimed at identifying distinctive mcT_2_motifs that best encapsulate the characteristics of the WM tissue. This preprocessing step is carried out prior to the optimization process expressed inEq. (2). The underlying principle of this approach is based on one key assumption: that*within the WM, there exists a finite set of microstructural configurations, each of which corresponds to a specific mcT_2_spectrum*. A flowchart of the algorithm is presented inFigure 1.

**Fig. 1.**
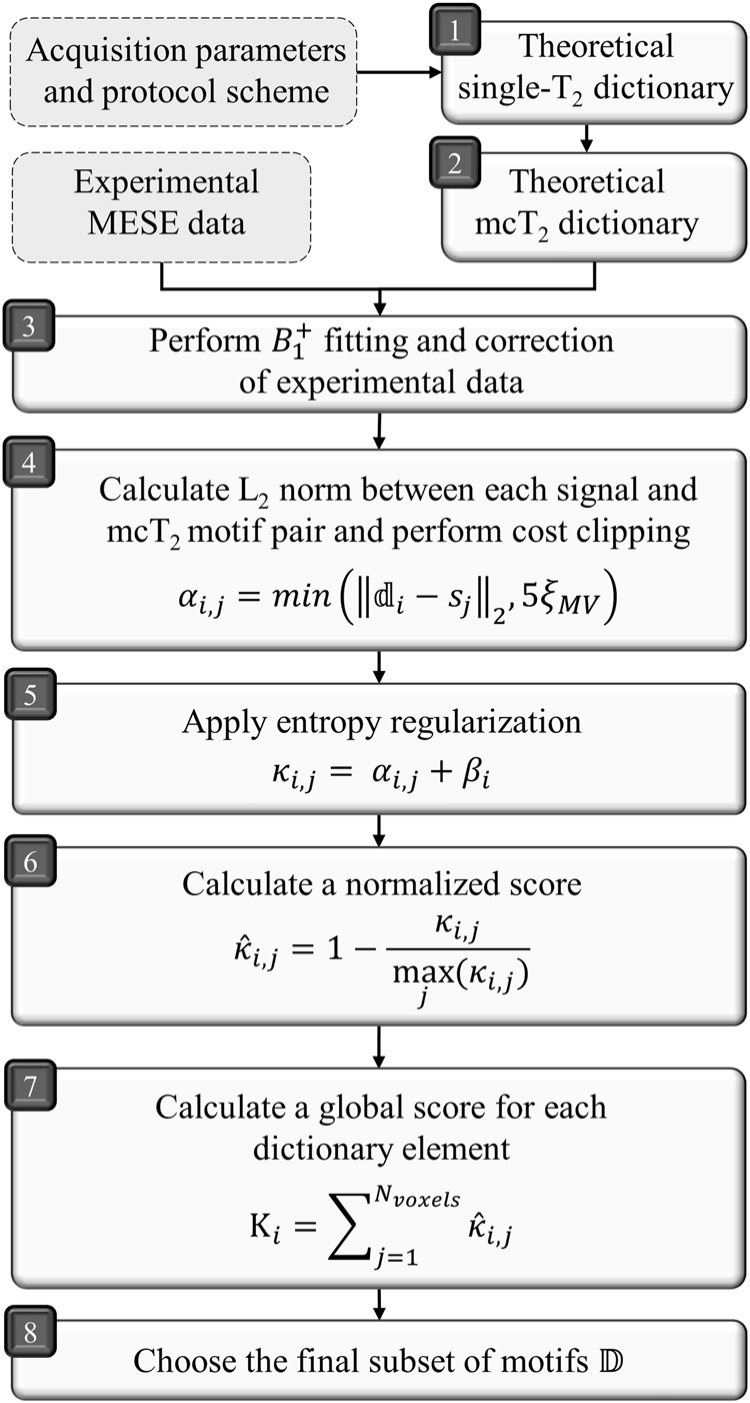
Data-driven algorithm flowchart. Input for the algorithm is marked with dashed frames. (Step 1) Generating single-T_2_dictionary using the EMC algorithm. (Step 2) Creating mcT_2_dictionary by combining single-T_2_signals with different fractions. (Step 3) Signal correction is performed for compensating for transmit field inhomogeneities. (Steps 4, 5) Statistical correlation is computed between each dictionary element$d_{i}$and signal$s_{j}$and added with entropy regularization to prevent overfitting. (Steps 6, 7) The result is then normalized and summed across all voxels to produce a global score. (Step 8) Select the basis elements with the highest scores while maximizing the orthogonality between motifs.

The input for the data-driven algorithm consists of MESE data, the exact pulse sequence scheme and acquisition parameters, and a WM mask. The algorithm consists of eight sequential steps. Step #1 involves the creation of a single-T_2_EMC dictionary of signals, spanning$N_{T_{2}}$logarithmically spaced T_2_values ranging from 10 to 800 ms. In step #2, a theoretical dictionary of all possible mcT_2_motifs is constructed by combining series of$N_{Comp}$single-T_2_signals, each with a relative fraction$f_{n}\in \left[0..1\right]$using a fraction resolution of$\Delta f=1/N_{frac}$, where$N_{frac}$is the number of discrete values that$f_{n}$can assume. Each simulated mcT_2_dictionary element*d*is expressed as

$$d=\sum_{n=1}^{N_{comp}}f_{n}\cdot d_{n}\;such\;that\sum_{n=1}^{N_{Comp}}f_{n}=1,$$ (3)

where$N_{comp}$is the number of compartments. Importantly, the number of compartments in the theoretical dictionary does not limit the number of compartments in the final mcT_2_spectrum for each voxel, which will comprise a linear combination of mcT_2_motifs. An important substage of step #2 involves pruning nonphysiological configurations from the mcT_2_dictionary, including mcT_2_motifs that lack a short T_2_component (<40 ms); motifs whose short T_2_component fraction exceeds 30%; and motifs that correspond to a single-T_2_value that is outside the range of T_2_values that exist in the WM region. This single-T_2_value of an mcT_2_motif is calculated by fitting the motif’s signal decay curve to a dictionary of single-T_2_signals. Stage #3 consists of correcting for transmit field (*B*_1_^+^) inhomogeneities, and is elaborated inSection 2.3.

In steps #4–5, statistical correlation is performed between each dictionary element$d_{i}$and every voxel signal$s_{j}$, by calculating a “cost”$\alpha_{i,j}$, defined as the L_2_norm difference between each pair. A regularization termβis added to this cost value to penalize motifs with high entropy, retrieving the simplest spectral features and reducing overfitting (L. Chen et al., 2002;Widjaja & Garland, 2005):

$$\kappa_{i,j}=\alpha_{i,j}+\beta_{i}$$ (4)

such that

$$\alpha_{i,j}={\left\|d_{i}-s_{j}\right\|}_{2}$$ (5)

$$\beta_{i}=\lambda_{Ent}\sum_{n=1}^{N_{comp}}-f_{i,n}\log\left(f_{i,n}\right),$$ (6)

where$f_{i,n}$is defined according toEq. (3). Prior to selecting the final basis set of motifs, we define a similarity criterion between a dictionary element ($d_{i}$) and voxel signal ($s_{j}$). This is used to apply cost clipping (Taal et al., 2011) for the purpose of reducing potential bias from motifs which are significantly different from the experimental data. The similarity threshold is denoted as$\xi_{MV}$and defined as

$$\xi_{MV}≜\sqrt{\delta_{MV}^{2}\cdot ETL},$$ (7)

where$\delta_{MV}$is set empirically. Each$d_{i}$and$s_{j}$pair is then considered similar if it obeys:

$$\alpha_{i,j}<\xi_{MV}.$$ (8)

In this process,$\alpha_{i,j}$is constrained by$\alpha_{i,j}\to \text{min}(\alpha_{i,j},$$5\xi_{MV})$, and$\kappa_{i,j}$is updated accordingly. In step #6, the$\kappa_{i,j}$scales are normalized to [0…1] range according to

$${\hat{\kappa }}_{i,j}=1-\frac{\kappa_{i,j}}{\underset{j}{\max}(\kappa_{i,j})}.$$ (9)

In step #7, the scores${\hat{\kappa }}_{i,j}$of each dictionary element$d_{i}$are summed across all voxels to yield a global score${Κ}_{i}$that expresses how well each motif matches the examined tissue:

$${Κ}_{i}=\sum_{j=1}^{N_{voxels}}{\hat{\kappa }}_{i,j}.$$ (10)

Finally, in step #8, a finite subset of motifs is selected, which will be used to separate each voxel’s signal into its underlying components. The selection process is based on two criteria: the highest${Κ}_{i}$score and a pseudo-orthogonality constraint which maximizes the differences between motifs in the final subset. A full step-by-step description of the selection process is detailed inAlgorithm 1.

**Table:** Algorithm 1

**Input:**	
$\left\{d_{1},d_{2},\ldots ,d_{N_{motifs}}\right\}$	Set of all mcT _2_ motifs sorted according to their score ${Κ}_{i}$
$\left\{T_{2}^{\left(d_{1}\right)},T_{2}^{\left(d_{2}\right)},\ldots ,T_{2}^{\left(d_{N_{motifs}}\right)}\right\}$	Single-T _2_ value, corresponding to each motif
$\hat{V}$	Set of all the voxels in the analyzed ROI
$V_{i}=\left\{j\;\text{|}a_{i,j}\;<\xi_{MV}\right\}$	Group of voxels that are similar to motif *i* (see Eq. (8) )

**Output:**	
D	A set of selected mcT _2_ motifs

**Algorithm:**	
1: D=∅ 2: $D\to D\cup d_{1}$ 3: $\hat{V}\to \hat{V}\backslash \text{​}V_{1}$	Initialize D to be an empty set. Add the mcT _2_ motif with the highest score to the set of selected motifs. Remove from the group of all voxels $\hat{V}$ , voxels that are similar to motif $d_{1}$
4: **for** $i=2,3\ldots ,N_{motifs}$ :	Loop over all remaining mcT _2_ motifs, sorted according to their score ${Κ}_{i}$
5: **if** $T_{2}^{\left(d_{i}\right)}\notin T_{2}\left\{D\right\}$ :	Check whether motif i does not have the same single-T _2_ value as any of the other motifs already in D
6: **if** $\hat{V}\cap V_{i}\neq \emptyset$ :	Check whether this motif holds new information, i.e., is it similar to voxels that were not yet accounted for by motifs already in * D *
7: $D\to D\cup d_{i}$	Add the motif to the set of selected motifs
8: $\hat{V}\to \hat{V}\text{​}\backslash V_{i}$	Remove from the group of all voxels $\hat{V}$ , voxels that are similar to motif i
9: **end if**	
10: **end if**	
11: **if** $\hat{V}=\emptyset$	If all voxels have been accounted for (i.e., $\hat{V}$ is an empty set)
12: **break for loop**	Break for loop and terminate the algorithm
13: **end if**	
14: **end for**	

After selecting the final set of motifsD, the signal is modeled as a linear combination of mcT_2_signals:

$$s=\sum_{i=1}^{\left|D\right|}W_{i}\cdot d_{i}=DW,$$ (11)

where$s\in {\mathbb{R}}^{ETL}$is the experimental signal,$W\;\in {\mathbb{R}}^{\left|D\right|}$is the unknown vector of weights of the elements inD, and|D|is the number of elements inD. The mcT_2_fitting problem now converts to solving for the unknown vectorWthrough a standard RNNLS optimization procedure:

$$\underset{W}{\text{argmin}}\frac{1}{2}{\left\|DW-s\right\|}_{2}^{2}+\lambda_{Tik}{\left\|W\right\|}_{2}^{2}+\lambda_{L_{1}}{\left\|W\right\|}_{1},\;\;\;such\;that\;W_{i}\geq 0,$$ (12)

which is similar toEq. (2), albeit with a modified encoding operator that has been learned from the tissue being analyzed. Notably, the result obtained fromEq. (12)isW, although our objective is to derive vectorw. Recalling that each motif is defined by a weights vectorf(Eq. (3)), and denoting the matrix of all weights as$F\text{​}\in {\mathbb{R}}^{N_{T_{2}}\times \;\left|D\right|}$, the final T_2_spectrum at each voxel will be

w=FW. (13)

Finally, MWF values are calculated from the$T_{2}$spectrum of each voxel as the relative energy between 0 and 40 ms, and the energy of the entire spectrum.

### 2.3 Correcting for transmit (B _1_^+^ ) field inhomogeneities

The extensive utilization of RF refocusing pulses in MESE signals can introduce bias due to transmit field inhomogeneities. To address this, the data-driven algorithm has been added with a preprocessing stage designed to estimate the$B_{1}^{+}$profile and subsequently correct the experimental data. As each theoretical EMC decay curve$d_{i}$depends on both$T_{2}$and$B_{1}^{+}$, the same holds for each mcT_2_motif which can now be expressed as$d(B_{1}^{+})$. Thus, each motifdtransitions from a 1D to 2D by adding a$B_{1}^{+}$dimension, discretized across$N_{B_{1}^{+}}$values in the range 80–120% (where 100% represents a fully homogeneous field).

$$d\left(B_{1}^{+}\right)=\sum_{n=1}^{N_{comp}}f_{n}\cdot d_{n}(B_{1}^{+})\;such\;that\sum_{n=1}^{N_{Comp}}f_{n}=1.$$ (14)

The$B_{1}^{+}$correction procedure involves three stages. First, the initial$B_{1}^{+}$profile is calculated for each voxel*j*by finding the dictionary motif$d(B_{1}^{+})$that has the lowest L_2_-norm difference to the experimental signal$s_{j}$. This is done using an exhaustive search over all dictionary elements and produces an initial solution for the transmit field map$B_{1,n\;=\;0}^{+}$. In the second stage, the field map undergoes iterative spatial smoothing within a region surrounding each voxel by minimizing the following cost function:

$${\text{B}}_{1,\text{n}+1}^{+}(j)=\underset{B_{1}^{+}}{\text{argmin}}\left[{\left\|d\left(B_{1}^{+}\right)-s_{j}\right\|}_{2}+\frac{\mu }{\left|N_{k}\right|}\sum_{r\in N_{k}}\left|B_{1}^{+}-B_{1,n}^{+}(r)\right|\right].$$ (15)

Here,$B_{1,n}^{+}$and$B_{1,n+1}^{+}$denote the transmit field profile at iteration n and n + 1, respectively, and$B_{1}^{+}$denotes the simulated range of$N_{B_{1}^{+}}$transmit field profile values. The cost function employed in this optimization consists of two components: first, a likelihood term that is responsible for finding the$B_{1}^{+}$value that best aligns with the data from voxel$s_{j}$; and second, a prior that imposes spatial smoothness within a 2D kernel using L_1_norm. The utilization of the L_1_norm in this process has been shown to enhance resilience against noise and outliers across various signal processing applications (Brooks et al., 2013;Markopoulos et al., 2014). μ represents the regularization weight,$N_{k}$denotes all voxels within the 2D kernel surrounding voxel$s_{j}$, and$\left|N_{k}\right|$is the number of voxels in$N_{k}$. The iterative process is terminated either when there is no change in the$B_{1}^{+}$value between iterations or when the number of iterations exceeds the predefined limit of$N_{iter}\;=200$. The resulting transmit field map is then denoted as$B_{1,opt}^{+}$.

Finally, the$B_{1,opt}^{+}$map is used to correct signal$s_{j}$in each voxel. Taking the mcT_2_motif with the optimal$B_{1}^{+}$value$d(B_{1,opt}^{+})$, the corrected signal$s_{j,cor}$is calculated as

$$s_{j,cor}\left(t\right)=s_{j}\left(t\right)\cdot \frac{d\left(t\right)}{d\left(B_{1,opt}^{+},t\right)},$$ (16)

where*d*denotes the “homogeneous” mcT_2_motif, corresponding to$B_{1}^{+}$value of 100%.

## 3 Methods

### 3.1 Validation on a numerical phantom

To evaluate the suggested method, numeric simulations of a 2D MESE protocol were performed on a Shepp–Logan phantom, using matrix size = 90 x 90, ETL = 11, echo time (**TE**) = 12 ms, interecho spacing = 12 ms, and bandwidth = 200 Hz/Px. Simulations were repeated for five tissue types, varying by the number of compartments ($N_{Comp}\;=1,2,3$), the myelin fractions, the relaxation times, and relative fractions of the intra-/extracellular water pools, and the$B_{1}^{+}$field profile. Detailed description of simulated mcT_2_configurations is delineated inFigure S1.

To benchmark the myelin mapping algorithm across different SNRs, Rician noise was introduced to the simulated signals at SNRs of 500, 300, 200, 100, and 50, defined as the ratio between the first echo amplitude and the standard deviation of the noise. mcT_2_fitting was performed according to the algorithm described inFigure 1, starting with generating an mcT_2_dictionary. The number of possible configurations ($N_{mcT_{2}}$) grows exponentially with$N_{T_{2}}$,$N_{frac}$, and*$N_{B_{1}^{+}}$*, which are used to construct the dictionary, and can be calculated using combinatorics. For example, for a choice of two compartments, this number will be

$$N_{mcT_{2}}=\left[\left(\begin{array}{c} N_{T2}\\ 1 \end{array}\right)+\left(\begin{array}{c} N_{T2}\\ 2 \end{array}\right)\left(N_{frac}\;-2\right)\right]\cdot N_{B_{1}^{+}},$$ (17)

which comes up to 3,404,700 elements for$N_{T_{2}}\;=200$,Δf=0.05,$N_{B_{1}^{+}}\;=9$($B_{1}^{+}\;=80:5:120\;\%$). The regularization weights$\lambda_{Tikh}$,$\lambda_{L_{1}}$, and$\lambda_{ent}$were determined through an exhaustive search in the range of 0–10, optimized for maximal MWF accuracy. The importance of choosing the right$\lambda_{Ent}$is further demonstrated inFigures S2 and S3, for SNRs of 500 and 100, respectively.

The minimization problem inEq. (12)was solved using MATLAB’s (Mathworks, version 2022b) Quadratic programming (seeAppendix Afor detailed description). To assess the algorithm’s stability, mean absolute error was computed as a function of$\lambda_{Tikh}$and$\lambda_{L_{1}}$values, for the five tissue types in the numeric phantom. Optimal reconstruction parameters for the conventional and data-driven approaches, i.e., those which yielded the highest accuracy, are detailed in the 3rd and 6th columns ofTable 1, respectively.

**Table 1.** List of optimal parameters for the conventional and data-driven approaches.

Parameter	Symbol	Conventional			Data driven		
		Numerical phantom	Physical phatnom	*In vivo*	Numerical phantom	Physical phatnom	*In vivo*
Number of single-T _2_ values	N _T2_	200	200	200	200	200	200
Range of single-T _2_ values		10…800	5…800	10…800	10…800	5…800	10…800
Fraction resolution	∆f	—	—	—	0.05	0.05	0.05
Short T _2_ fraction limit [%]		—	—	—	30	30	30
Similarity parameter	δ _MV_	—	—	—	0.01…0.02 *	0.008	0.01
Entropy regularization	λ _Ent_	—	—	—	0.001	0.05	0.001
Tikhonov regularization	λ _Tikh_	0.1	0.0005	0.1	0.001	0.0005	0.001
L _1_ regularization	λ _L1_	0.01	0.01	0.01	0.01	0.01	0.01
Regularization for B _1_^+^ correction	μ	1	None	1	1	None	1

* Depends on SNR.

### 3.2 Validation on a physical phantom

An mcT_2_phantom was prepared using MnCl_2_solutions at concentrations of 0.11, 0.15, and 0.6 mM, producing T_2_relaxation times of 80, 60, and 20 ms, respectively. Nine tubes, each with a volume of 70 ml, were used as containers of a physical multicompartment phantom, featuring three distinct internal compartments. The first compartment consisted of background solution with T_2_of 80 ms. Varying number of 3 and 5 mm capillary tubes were then inserted into the container tubes, filled with 60 and 20 ms solutions, and serving as the 2nd and 3rd compartments. This resulted in nine relative fractions of the short T_2_(20 ms) compartment, equal to 0%, 0%, 3.8%, 7.1%, 11.1%, 14.3%, 18.5%, 21.7%, and 26.2%. Full description of the nine different multicompartment configurations is delineated inTable S1.

MRI scans were conducted separately for each tube using a 2D MESE protocol with the following parameters: TE = 7.9, repetition time (TR) = 5000 ms, interecho spacing = 7.9 ms, ETL = 24, FOV = 750 x 750 mm^2^, matrix size = 30 x 30, slice thickness = 3 mm, N_Slices_= 10, bandwidth = 399 Hz/Px, and total scan time of T_acq_= 2:45 min. A key aspect of these scans is the huge voxel size = 25 x 25 mm^2^, ensuring that each*tube was fully encapsulated within a single voxel*. This unique setup enabled the generation of genuine, experimental mcT_2_signals, with known ground truth fractions of the short T_2_component. To generate a large number of mcT_2_signals, each low-resolution scan was repeated four times with varying slice offsets. Lastly, to assess the interscan repeatability of the fitting process, two of the tubes having fractions of 7.1% and 18.5% were scanned twice, resulting in an overall number of 440 separate signals (10 slices x 4 offsets x 11 tubes).

Postprocessing and statistical analysis of the phantom data included the determination of ground truth MWF values based on the relative area of the short T_2_tubes, derived directly from the phantom geometry. The data-driven analysis utilized data from all tubes, creating a diverse dataset, with a range of single-T_2_values that is similar to what is expected*in vivo*. Mean and standard deviation (**SD**) of the MWF were calculated for each tube, and Pearson correlation and root mean square difference (**RMSD**) were computed to assess the agreement with the ground truth MWF values. Interscan repeatability was assessed by calculating RMSD and correlation coefficient, and performing Bland–Altman analysis for the two tubes which were scanned twice. Detailed list of all postprocessing parameters which gave the highest accuracy for both approaches is outlined inTable 1, 4th and 7th columns.

### 3.3 MWF mapping of healthy subjects

A group of 26 healthy subjects (39.2 ± 5.5 y/o, 15 males) was scanned on a 3T Prisma MRI scanner (Siemens Healthineers), after signing an informed consent and under Helsinki approval by Sheba Medical Center (3933-17-SMC). MRI scans included a 2D MESE protocol with TE/TR = 12/5000 ms, interecho spacing = 12 ms, ETL = 11, FOV = 192 x 156 mm^2^, matrix size = 192 x 156, slice thickness = 3 mm, N_slices_= 32, acquisition bandwidth = 200 Hz/Px, GRAPPA acceleration factor = 2, and T_acq_= 7:35 min. 3D T_1_-weighted magnetization prepared two rapid gradient echo (**MP2RAGE**) data were collected for brain segmentations using TE/TR = 3.5/4000 ms, TI = 732 and 2220 ms, FOV = 224 x 168 mm^2^, matrix size = 224 x 168, slice thickness = 1 mm, N_slices_= 192, and T_acq_= 6:24 min.

Prior to MWF fitting, MESE data were denoised to enhance SNR using the MP-PCA algorithm (Stern et al., 2022), following a previous study that demonstrated the benefits of denoising for multicomponent relaxometry fitting (Does et al., 2019). Extraction of WM mask was performed on the MPRAGE images, followed by registration to MESE image space using Freesurfer tools (Fischl, 2012;Reuter et al., 2010). MWF maps were generated using the conventional and data-driven techniques, using the postprocessing parameters detailed inTable 1, 5th and 8th columns, and a fixed kernel size of 15 x 15 mm^2^for$B_{1}^{+}$correction. Single-T_2_maps were also calculated based on the mcT_2_spectrum within each voxel by (*i*) generating a single-T_2_signal using a linear combination of all T_2_components (seeEq. (11)), and (*ii*) performing single-T_2_fitting using the EMC algorithm (Radunsky et al., 2021). Reproducibility of the data-driven MWF fitting algorithm was tested using scan–rescan available data from 22 out of the 26 healthy subjects. The time difference between the two scans was 30 ± 13 days.

### 3.4 MWF mapping in people with MS

A cohort of 29 individuals with relapsing–remitting MS (44.2 ± 11.8 y/o, 9 males) was scanned on a 3T scanner at Sheba Medical Center after providing informed consent and under Helsinki approval (6923-20-SMC). MRI scan included a 2D MESE protocol [TE/TR = 12/4600 ms, interecho spacing = 12 ms, ETL = 11, FOV = 192 x 220 mm^2^, matrix size = 112 x 128, slice thickness = 3 mm, N_slices_= 33, GRAPPA acceleration factor = 2, bandwidth = 200 Hz/Px, T_acq_= 5:15 min]; 3D T_1_w MPRAGE [TE/TR = 2.3/1800 ms, TI = 900 ms, FOV = 256 x 256 mm^2^, matrix size = 256 x 256, slice thickness = 1 mm, N_slices_= 176, T_acq_= 4:35 min]; fluid attenuated inversion recovery (**FLAIR**) [TE/TR = 83/9000 ms, TI = 2500 ms, FOV = 240 x 195 mm^2^, matrix size = 320 x 260, slice thickness = 2 mm, N_slices_= 64, total scan time = 4:30 min]. MWF maps were generated using the same parameters as for the healthy subjects. T_2_maps were calculated from the mcT_2_spectrum at each voxel, following a procedure described above for the healthy subjects.

### 3.5 Statistical analysis

Mean and SD of MWF values were calculated using the data-driven approach for six manually segmented 2D normal-appearing WM (**NAWM**) regions. These included the genu of the corpus callosum (**GCC**), splenium of the corpus callosum (**SCC**), frontal lobe, temporal lobe, occipital lobe, and the entire WM (segmented automatically using Freesurfer software). Illustration of the segmented ROIs is shown inFigure S4. Scan–rescan measurements were assessed for repeatability by calculating correlation coefficient and performing Bland–Altman analysis.

To compare MWF values between healthy subjects and people with MS, a two tailed unpaired*t*-test with significance level of$\alpha_{significance}=0.001$was performed. Bonferroni multiple comparisons correction was applied by dividing$\alpha_{significance}$by the number of tests, which was 6 in this case. Classification of people with MS vs. healthy controls was performed based on MWF values at each normal-appearing region, and receiver operating characteristic (**ROC**) curves were generated for each ROI, followed by calculating the area under the curve (**AUC**) as a metric of classification performance.

## 4 Results

The performance of the data-driven fitting of numerical phantom data is shown inFigure 2along with a comparison with the conventional RNNLS approach. Mean absolute errors of 0.2%, 0.5%, 0.7%, 1.2%, and 1.8% were produced by the data-driven approach for SNRs of 500, 300, 200, 100, and 50, respectively. Conventional fitting, in contrast, showed consistently higher mean absolute errors of 2.4%, 2.5%, 2.5%, 2.8%, and 3.7% across the tested SNR values.$B_{1}^{+}$bias field correction using the data-driven approach also showed high correlation to ground truth values with mean absolute errors of 0.1%, 0.1%, and 0.4% for SNRs > 100, while the conventional approach struggled to estimate$B_{1}^{+}$accurately and produced mean absolute errors of 6.0%, 5.1%, and 4.7%. At SNRs ≤ 100, higher deviations were observed for the$B_{1}^{+}$maps using both approaches with mean absolute errors of 2.8% and 5.5% for the data-driven technique and 5.3% and 6.1% for the conventional fitting at SNRs 100 and 50, respectively.

**Fig. 2.**
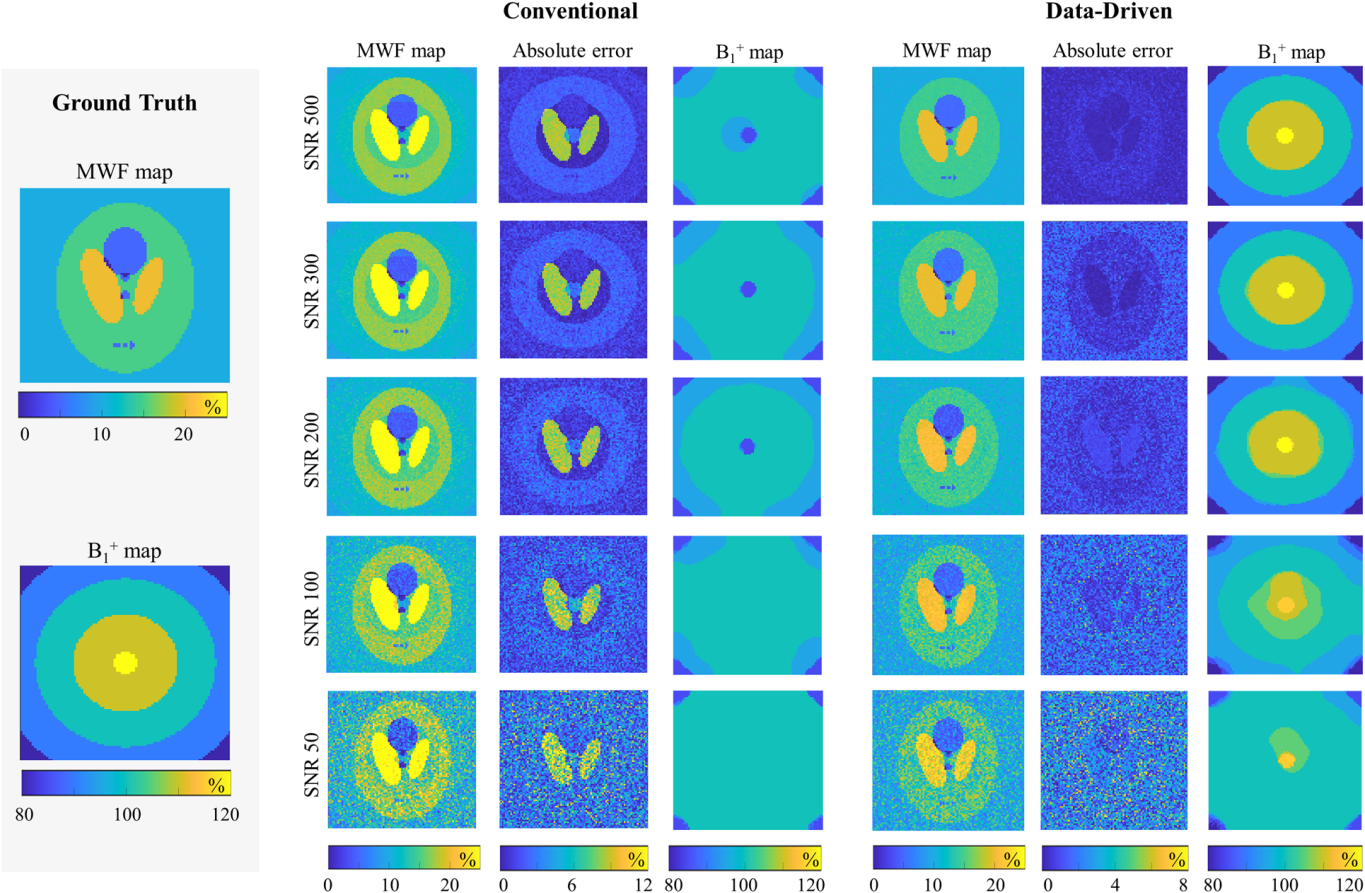
MWF mapping in a numerical phantom. Ground truth MWF and$B_{1}^{+}$maps are presented in the left panels. Right panels illustrate the performance of conventional and data-driven fitting approaches at different SNRs. Figure shows fitted MWF maps, absolute error maps, and reconstructed$B_{1}^{+}$profiles.

InFigure 3, stability maps are provided, depicting conventional and data-driven fitting performance across$\lambda_{Tikh}$and$\lambda_{L_{1}}$regularization values and different SNRs. The data-driven approach exhibits consistently small mean absolute errors over a wide range of L_1_and Tikhonov regularization weights for all tested SNRs. In contrast, the conventional approach demonstrates large mean absolute errors throughout the entire range of regularization settings.

**Fig. 3.**
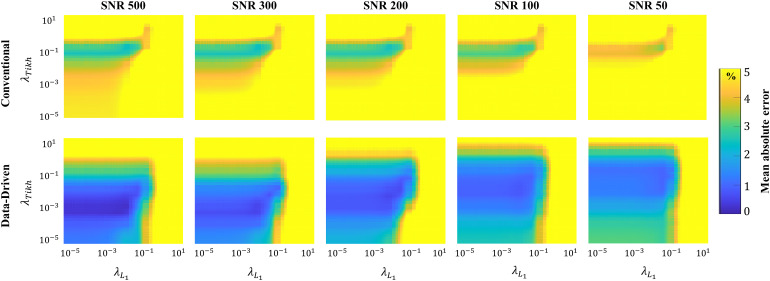
Stability of the conventional and data-driven MWF fitting approaches showing the mean absolute error (in %) as function of$L_{1}$and Tikhonov regularization weights for numerical phantom across different SNRs.

The design of the three-compartment physical phantom is shown inFigure 4A-B. Fitted MWF versus ground truth fractions using the data-driven technique are shown inFigure 4C, exhibiting high linear correlation (r) and small absolute error of 0.83 ± 0.51%. Similar analysis of conventional mcT_2_fitting is shown inFigure S5.

**Fig. 4.**
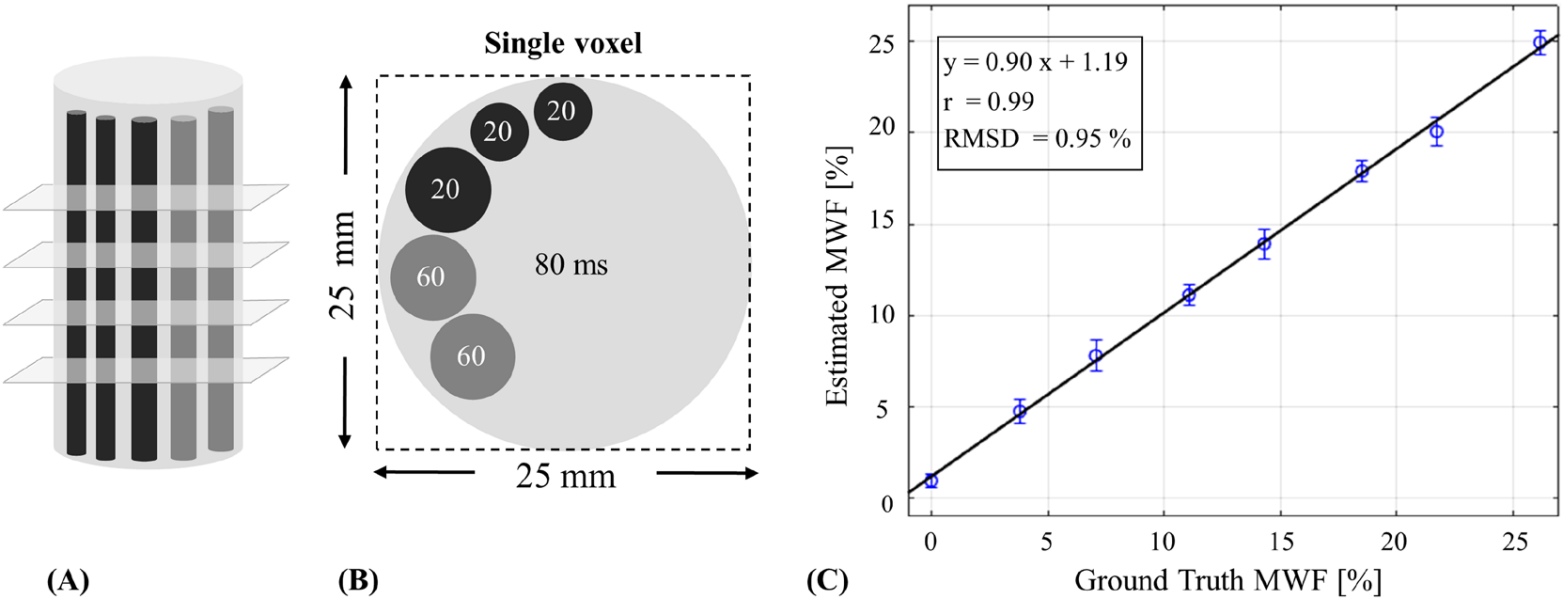
Sagittal (A) and axial (B) illustrations of the unique multicompartment phantom design used in the study. (C) Correlation between data-driven and ground truth MWF values. Intratube variability (SD) of MWF values is shown as error bars for each tube. Black line denotes the best fit line. Correlation value of r = 0.99, RMSD value of 0.95%, and mean absolute error of 0.83 ± 0.51% were achieved between the estimated and ground truth MWF values.

Scan–rescan analysis of the physical phantom is presented inFigure 5A-Bfor two tubes with MWF fractions of 7% and 18.5%. The data-driven approach exhibits small RMSD of 0.76% and 0.22% for the 7% and 18.5% tubes, respectively. Further statistical analysis is presented using the Bland–Altman plots inFigure 5C-D, demonstrating a good agreement between the scans with difference of 0.40 ± 0.66% and 0.15 ± 0.16% for the 7% and 18.5% tubes, respectively. Similar analysis of conventional mcT_2_fitting is shown inFigure S6.

**Fig. 5.**
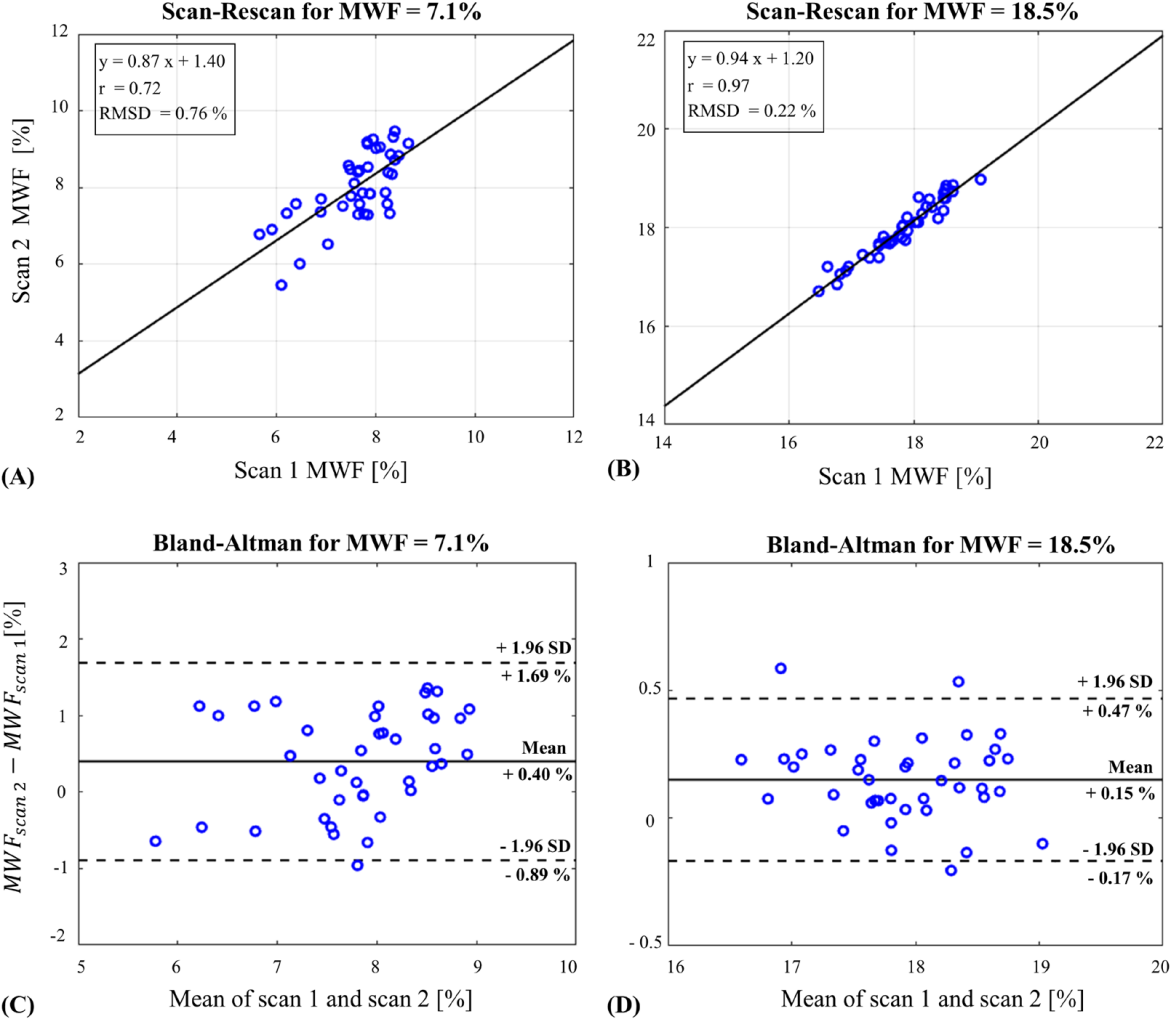
Scan–rescan analysis and Bland–Altman plot for MWF values derived using the data-driven approach for two tubes containing (A, C) 7.1% and (B, D) 18.5%.

T_2_-weighted images, T_2_maps, and MWF maps from three representative healthy subjects are presented inFigure 6. No correlation was observed between the MWF and T_2_values, i.e., higher MWF values do not necessarily indicate lower T_2_values as can be seen in the GCC and SCC regions.*In vivo*repeatability of MWF maps was assessed using scan–rescan data from 22 healthy subjects, summarized in the Bland–Altman and correlation plots in Figure7. The data-driven approach yielded realistic MWF values and demonstrated good repeatability, with a correlation coefficient of 0.91 and RMSD of 1.08%. Similar analysis of conventional mcT_2_fitting for healthy subjects is shown inFigures S7-S8.

**Fig. 6.**
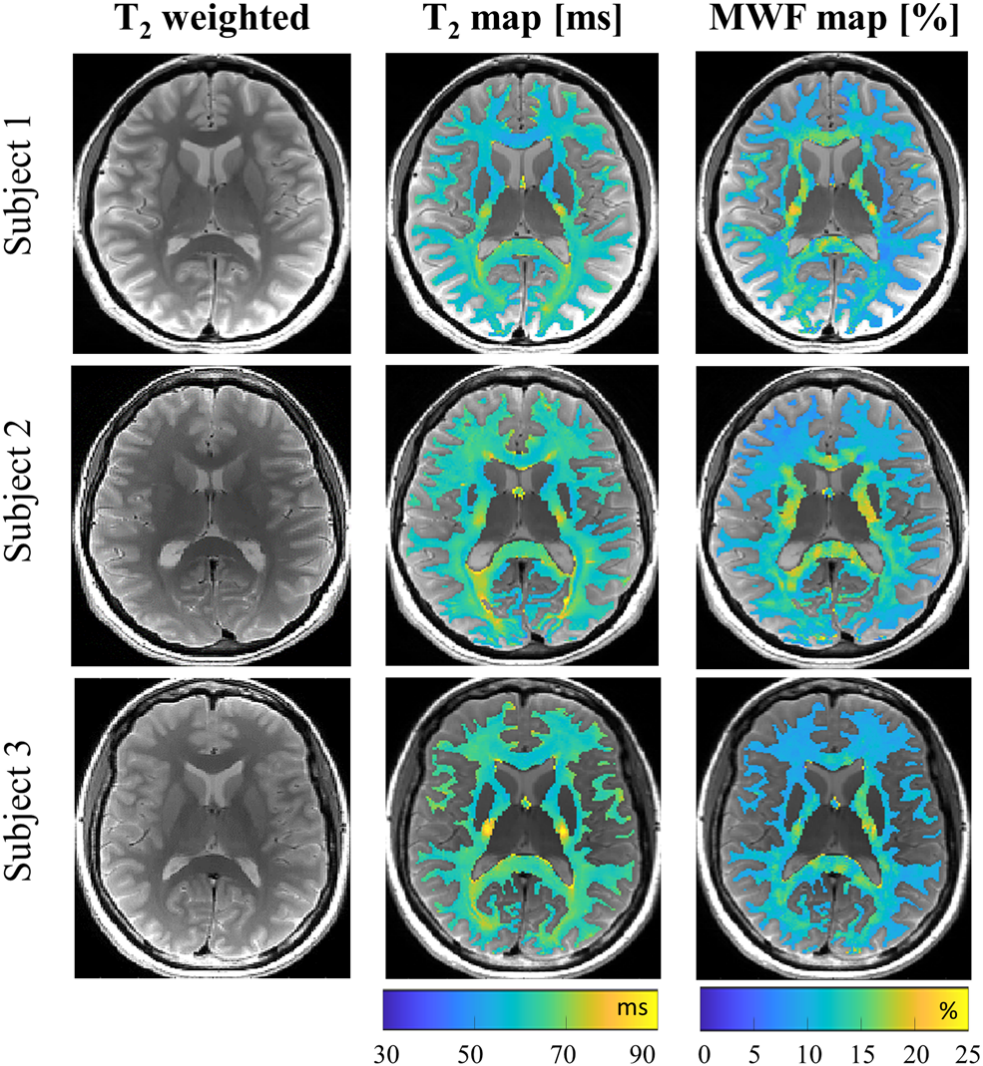
T_2_-weighted images, T_2_maps, and MWF maps for three healthy subjects, generated using the data-driven fitting approach.

**Fig. 7.**
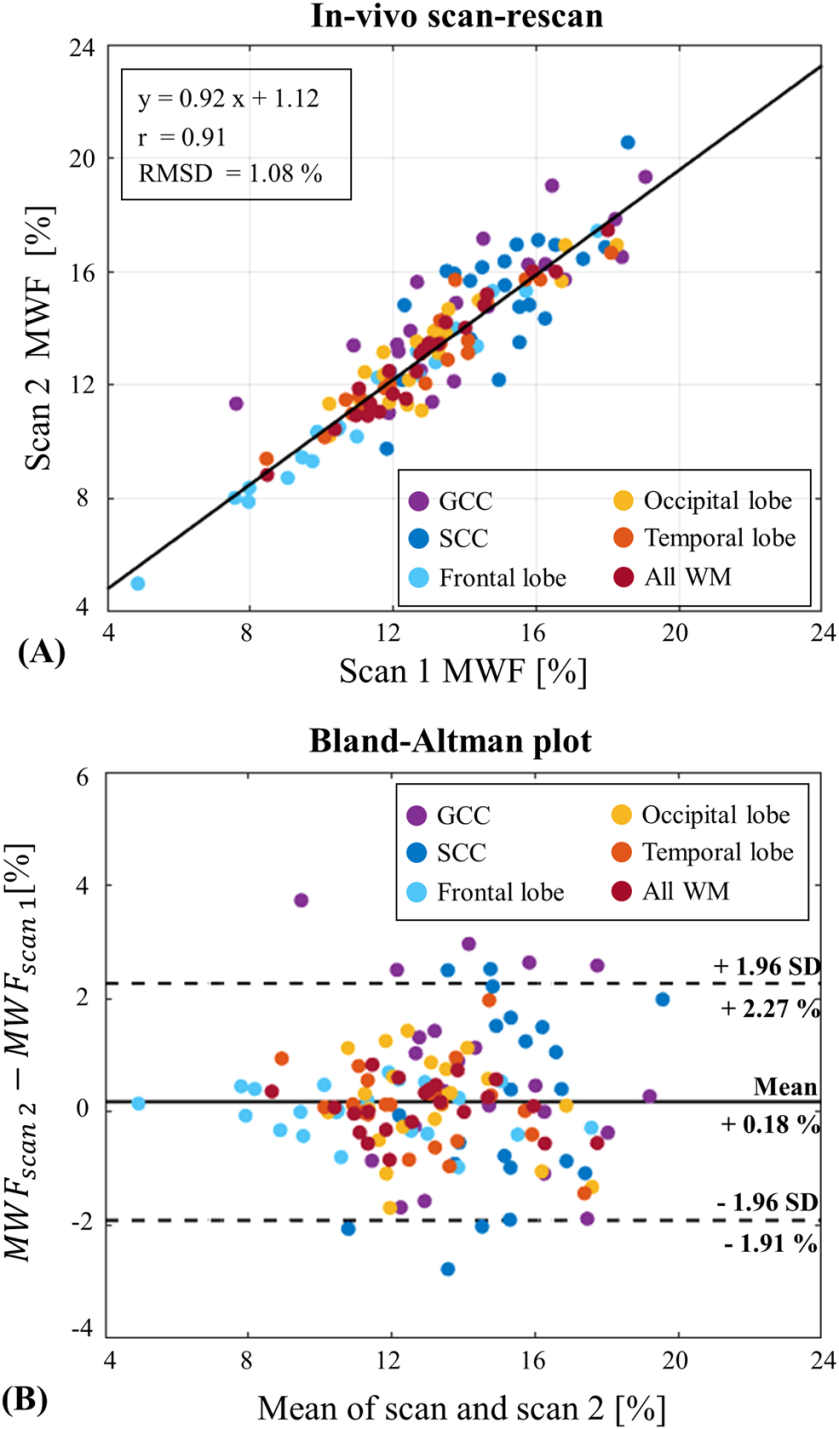
(A) Scan–rescan correlation analysis and (B) Bland–Altman plot for*in vivo*MWF values derived using the data-driven algorithm.

**Fig. 8.**
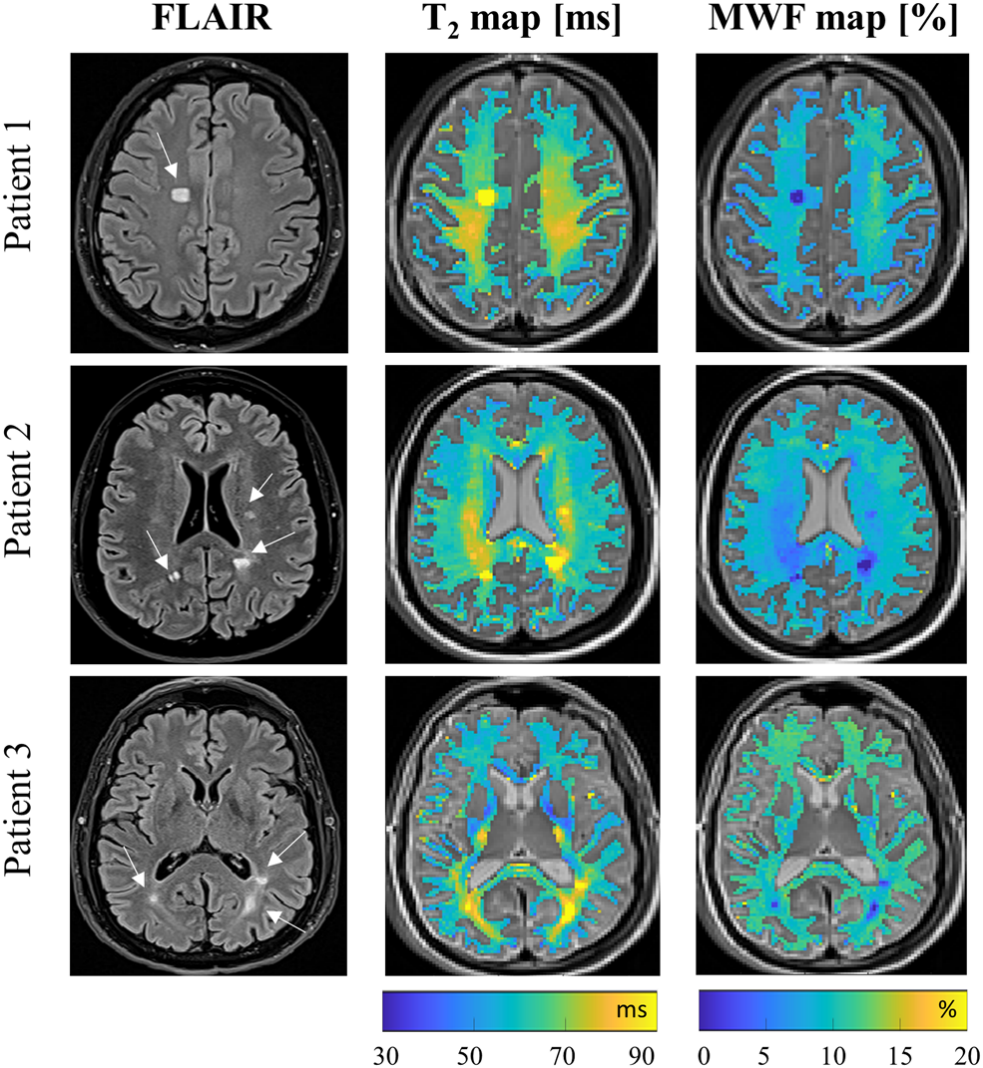
FLAIR images, T_2_maps, and MWF maps for three people with MS fitted using the data-driven approach.

Figure 8shows FLAIR images, T_2_maps, and MWF maps of three representative people with MS. The advantage of the data-driven approach is clearly demonstrated in this figure, showing how*quantitative*maps reveal subtle changes which are indicative of inflammation and demyelination within NAWM, and which are not visible in the*qualitative*FLAIR images. Similar quantitative maps produced using the conventional mcT_2_fitting are shown inFigure S9.

MWF values for specific brain ROIs were extracted for both healthy subjects and people with MS, and then used to classify subjects between these groups. Box plots of the extracted values are illustrated inFigure 9, along with classification ROC curves. The data-driven approach produced a significant difference (p-value < 0.0001) in mean MWF between healthy subjects and people with MS across all ROIs, with a relative reduction in MWF ranging from 20% to 38%. Full numeric values per ROI are given inTable S2. The data-driven approach yielded consistently higher AUC for all tested regions. Similar analysis using the conventional mcT_2_fitting is shown inFigure S10andTable S3.

**Fig. 9.**
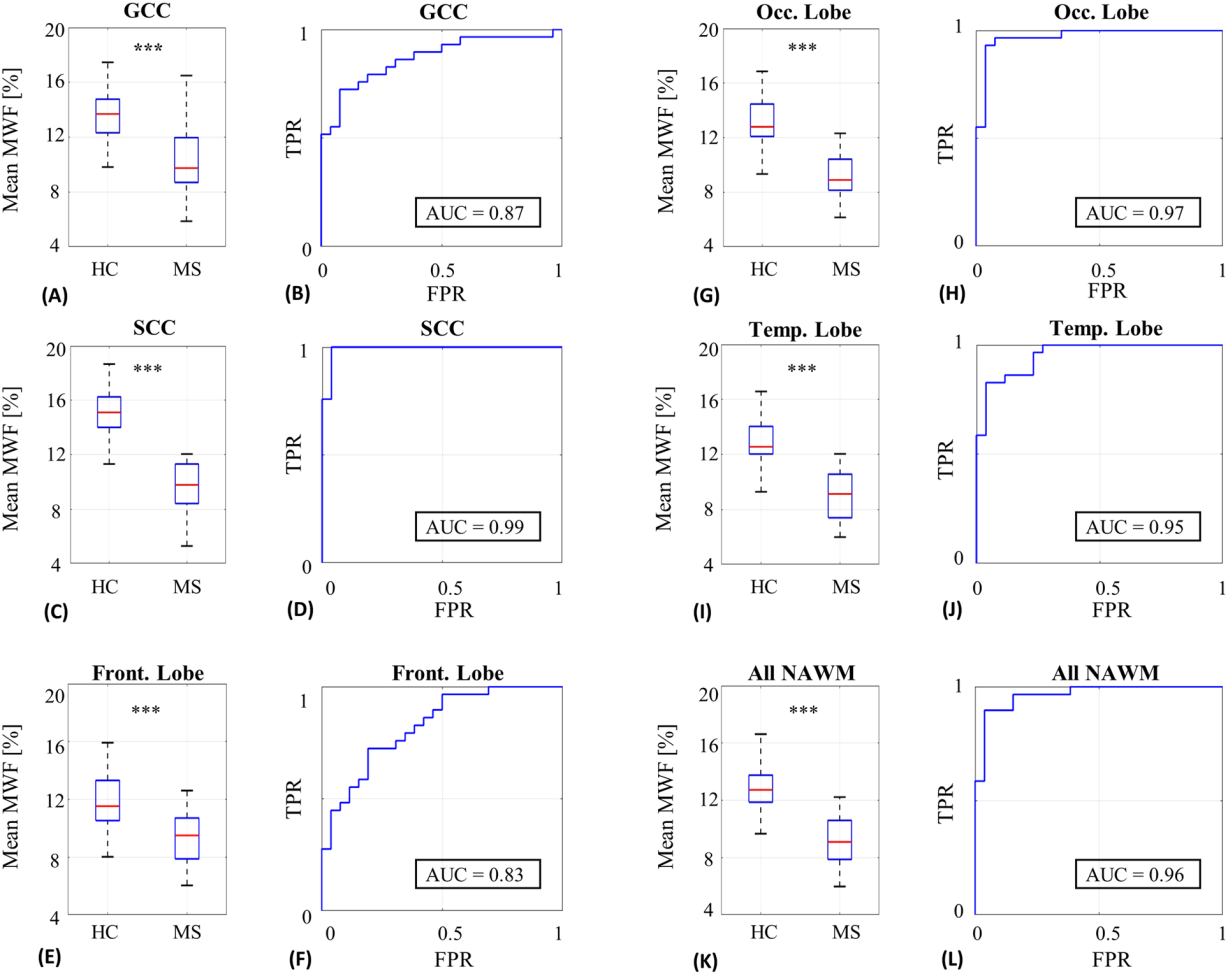
Box plots of MWF values for six WM ROIs, comparing healthy controls (**HC**) and people with MS. Statistically significant separation is achieved between the two populations for all tested ROIs (***p-value< 0.0001) after correcting for multiple comparisons. ROC curves are shown on the 2nd and 4th columns, calculated based on mean MWF values in NAWM only (i.e., excluding lesions). (A-B) Genu of corpus callosum (GCC). (C-D) Splenium of corpus callosum (SCC). (E-F) Frontal (Front.) lobe. (G-H) Occipital (Occ.) lobe. (I-J) Temporal (Temp.) lobe. (K-L) All NAWM.

## 5 Discussion

The significant ambiguity within mcT_2_search space poses a substantial obstacle for achieving reliable MWF values. This study introduces several improvements to a recently developed MWI technique based on mcT_2_analysis of MESE data, with corrections for transmit field inhomogeneities. The method begins by identifying global mcT_2_motifs of the entire tissue, which are then used for localized signal analysis at each voxel, while accounting for local variations in the$B_{1}^{+}$field. As an initial step, a comprehensive dictionary of mcT_2_signals is generated, tailored to match the precise pulse-sequence parameters of the MESE protocol. Extraction of specific pulse-sequence parameters is necessary in order to use the EMC model of MESE signals, which stands at the basis of the data-driven MWF mapping algorithm. On Siemens scanners, this is done via the IDEA software. Accessing these parameters requires proprietary scanner code and a research agreement with the corresponding vendor.

The identification of tissue-specific mcT_2_motifs significantly reduces the number of potential solutions, thereby alleviating the intrinsic ill-posed nature of mcT_2_analysis. The primary objective of this stage is to keep the most relevant motifs—either having good correlation to a large number of voxels or very good correlation with a limited number of voxels, like lesions. The ensuing set of motifs constitute a pseudo-orthogonal basis set with maximum information about the tissue. This strategy mitigates the risk of converging toward less physiological solutions, even if they might demonstrate higher fitting accuracy at the voxel level. Although this paper primarily focused on 2D MESE sequences which can be run at clinical scan times of 5–8 minutes (depending on image resolution), the data-driven approach can also be adapted for use with 3D MESE sequences using dictionaries of exponentially decaying signals. Recently, another data-driven approach has been suggested (Piredda et al., 2022), where different regression models are trained using single relaxometry measurements (such as single T_1_and single T_2_) to predict MWF values. While this approach focuses on establishing a generalized model for MWF prediction across acquired data, the method presented herein is based on learning specific characteristics of the WM of each individual. Similarly, the proposed approach has the potential to extend its applicability by conducting the data-driven analysis on a collective group of individuals. However, such generalization should be approached cautiously, considering various factors such as age, gender, and other cofactors that may affect myelination patterns.

The number of compartments used to generate the simulated mcT_2_dictionary may be either two, three, or even higher if sufficient computational resources are available. Here, we conducted tests with both two- and three-compartment dictionaries and determined that a two-compartment dictionary is sufficient and does not introduce higher errors in the measured MWF, while allowing to perform analysis on a standard PC. Importantly, the number of compartments in the mcT_2_dictionary does not impose any constraints on the final number of components in the T_2_spectrum, as there is no inherent limit on the number of mcT_2_motifs used to represent the signal in each voxel (seeEq. (11)).

The range of single-T_2_values used to filter nonphysiological mcT_2_motifs can exhibit variations among subjects and may be wider in tissues containing pathologies such as MS lesions. It is, therefore, crucial that the range and dynamic resolution of T_2_values inDbe sufficiently dense within the physiological range of T_2_values to construct an mcT_2_dictionaryDthat will faithfully characterize the tissue. Various choices of$N_{T_{2}}$have been reported in the literature (Kumar et al., 2018;Nagtegaal, Koken, Amthor, De Bresser, et al., 2020;Prasloski, Mädler, et al., 2012;Whittall & MacKay, 1989). One study reported that myelin quantification remains unaffected by the choice of$N_{T_{2}}$when it is sufficiently large, while a small$N_{T_{2}}$can result in substantial variations in MWF values (Kumar et al., 2016,^2018^). In the current study, we chose$N_{T_{2}}$of 200 to maintain consistency with the number of single-T_2_values used in creating the mcT_2_dictionary. The ability to generate a large mcT_2_dictionary in our study was made feasible by implementing a subsequent dilution process. This crucial step served to tailor the mcT_2_basis functions to match the physiological mcT_2_configurations found in the tissue and avoid erroneous combinations that could potentially arise due to noise.

The numerical results demonstrate that the data-driven algorithm can produce consistently accurate results, even in scenarios with substantial variations in the range of T_2_values, short T_2_fractions, and high$B_{1}^{+}$inhomogeneity levels. The numerical phantom was designed to mimic physiological MWF values, SNR levels, and matrix sizes matching*in vivo*scan settings. Accordingly, similar regularizations were applied to the numerical phantom and*in vivo*data. In this study,$B_{1}^{+}$estimation relied on MESE data rather than a separate$B_{1}^{+}$mapping scan. Recent study showed that acquiring an independent$B_{1}^{+}$map can improve the MWF results (Mehdizadeh & Wilman, 2022). Nevertheless, the data-driven algorithm was able to produce relatively accurate$B_{1}^{+}$maps for all SNR values compared with the conventional approach, contributing to the accuracy of the final MWF maps. Further investigation is warranted to compare the reconstructed$B_{1}^{+}$maps using the data-driven approach with independently measured$B_{1}^{+}$maps. Another important conclusion, demonstrated in the numeric simulation, was that the values of$\delta_{MV}$, and consequently$\xi_{MV}$, should scale with SNR as higher noise levels require less stringent similarity criterion between simulated and experimental signals. Lastly, the stability maps presented in this study highlight the data-driven algorithm’s robustness across a range of$L_{1}$and Tikhonov regularization weights.

Findings from experiments conducted on the physical phantom provide valuable insights into the performance of the data-driven algorithm in a controlled setting. The unique design of the phantom used in this study provided valuable ground truth reference. Despite certain model limitations, such as the absence of exchange and diffusion effects, this phantom offered a genuine benchmark to assess the data-driven algorithm’s accuracy. The results demonstrate the data-driven algorithm’s reliability in terms of both accuracy and precision, and across a wide range of MWF values. Scan–rescan experiments further emphasized the strengths of the data-driven approach, revealing high repeatability, exhibited as low interscan RMSD in comparison with conventional processing. It is worth noting that different regularization parameters were chosen for the physical and numerical phantom data. This adjustment was necessary due to the variations in scan parameters and physical properties of each phantom, such as voxel size and the distribution of T_2_values. These differences were carefully considered to ensure accurate evaluations.

The data-driven myelin mapping tool exhibited high reproducibility also*in vivo*. Repeatability tests demonstrated high correlation and small RMSD, which aligns with previous studies (Levesque et al., 2010;Meyers et al., 2009). Examining MWF and T_2_maps versus the T_2_-weighted images from healthy subjects indicates that the intensity of qualitative T_2_-weighted images cannot be used as an accurate marker of myelin content, seeing as the intensity in qualitative images depends not only on myelin content, but also on other factors such as the total water content, macromolecular content, pathology, as well as on$B_{1}^{+}$inhomogeneities. Thus, from a radiological point of view, changes in T_2_weighting correspond more tightly with the tissue’s single T_2_, rather than MWF values.

Considering the comparison between the healthy subjects and people living with MS, data-driven MWF values were significantly different between healthy subjects and people with MS across all assayed NAWM ROIs, even after stringent correction for multiple comparisons. High AUC was furthermore achieved for all ROIs, indicating the potential of data-driven MWF values as a biomarker for MS. These findings align with previous studies (Faizy et al., 2016;Laule & Moore, 2018) and indicate a consistent reduction in MWF values in people with MS in comparison with healthy subjects. In contrast to the phantom experiments,*in vivo*scans lack ground truth MWF values. The processing of*in vivo*data thus used regularization weights derived from the numerical phantom experiments, as they shared the same scan settings.

MWF values were estimated in this study also using a conventional approach, which differs from the data-driven approach by using a theoretical dictionary of single-T_2_signals, rather than an mcT_2_dictionary of motifs which were learnt from the examined tissue. Corresponding MWF values were less accurate and precise for all assayed models. Numerical phantom results illustrated the conventional approach’s inability to produce accurate values, exhibiting substantial mean absolute error across the entire range of regularization settings. Conventional analysis of the scan–rescan data of the physical phantom (Fig. S6) showcased a wider spread of values, indicating increased sensitivity to random interscan signal variations and to noise vis-à-vis data-driven fitting. In the*in vivo*repeatability test (Fig. S8), the conventional approach exhibited similar correlation coefficient to the data-driven approach, but significantly higher MWF values across ROIs, similar to findings from the numerical simulations. Notably, this overestimation in healthy subjects persisted in people with MS as well (Fig. S9). Furthermore, in specific regions such as the frontal lobe and GCC, the conventional approach generated high AUC values albeit in an opposite direction to the natural progression of MS, showing*higher*MWF values for people with MS vs. controls (Fig. S10andTable S3).

The primary cause of this overestimation can be attributed to the notably high Tikhonov regularization value, which was larger by two orders of magnitude compared with the data-driven approach. As described inEq. (2), a higher Tikhonov regularization leads to a smoother spectrum, thereby necessitating the inclusion of more spectral components in the optimization process. Notably, conventional processing incorporated a broader range of T_2_values compared with the final set of selected motifs when using data-driven processing. It is important, however, to note that both data-driven and nondata-driven approaches produced identical T_2_maps, indicating that the center of mass of the spectra remained unchanged. The usage of a large Tikhonov regularization value causes selection of longer T_2_values, which was counteracted by increased energy in the short T_2_range. Utilizing a smaller regularization value (than the one which yielded the best results in the numerical simulations) would result in a significantly wider spread and lower quality in scan–rescan outcomes (result not shown).

Notwithstanding the successful results obtained using data-driven fitting, the current study has several limitations which should be considered. Firstly, the absence of ground truth*in vivo*limits one’s ability to validate the calculated MWF values. While correlations with histology can provided some insights into microstructural features (Laule et al., 2006), their applicability is constrained by postmortem tissue changes and the inherent variability in procedures such as fixation, slicing, and staining. Secondly, our proposed method, like other RNNLS-based approaches, operates under the assumption of a slow exchange regime, where the intercompartmental exchange is slow relative to the acquisition time (TE). Although previous reports suggested that exchange plays a minor role in MWF (Kalantari et al., 2011), there are also claims that the exchange of water between microenvironments may bias MWF values (Levesque & Pike, 2009), requiring to expand the tissue model to incorporate exchange, e.g., by using Bloch–McConnell equations (Harkins et al., 2012).

Thirdly, in our experiments, maximal TE of 132 ms was used. Optimal estimation of the T_2_values of slow relaxing intra-/extracellular water pools would, however, require longer echo trains, which are not feasible in clinical settings due to the increase in specific absorption rate (**SAR**) caused by the addition of refocusing pulses. Furthermore, longer ETLs would limit the number of slices that can be acquired within one TR, once again requiring the use of longer TRs and longer scan time. Requiring a coverage of at least 9 cm within clinically feasible scan times thus forces the use of maximal TEs of 120–150 ms, thereby trading off some of the encoding quality of the longer T_2_components. Nevertheless, this constraint is less limiting when mapping MWF values seeing as we are mostly interested in the short T_2_component (T_2_< 40 ms), while trading off some of the accuracy in the estimation of the long T_2_components should have lower influence on the resulting MWF values.

It is important to note that MWF only serves as a proxy for myelin content, while reduction in measured MWF can, e.g., result from either lower myelin content (demyelination) or higher intra/extra water content (inflammation or edema). Previous studies have suggested that the total water content has a minimal effect on MWF, with reductions primarily attributed to demyelination (Laule et al., 2004;Vavasour et al., 2021). Another factor that could influence the measured MWF is iron content, which is known to decrease T_2_relaxation time and may lead to overestimation of MWF (Birkl et al., 2019). Contrasting findings, however, have been reported, as another study demonstrated lower MWF values in regions with higher iron content (Khattar et al., 2021), suggesting that further investigation is needed to gain deeper insights into this cofactor. Additionally, incorporating other imaging contrasts alongside mcT_2_, such as diffusion (Kolind et al., 2008;Rahmanzadeh et al., 2021) and magnetization transfer (Schmierer et al., 2004), may provide additional valuable information and more accurate estimation of microstructural tissue changes.

## 6 Conclusions

This study introduces a new data-driven approach to mcT_2_analysis, which includes a new B_1_^+^correction procedure and incorporates entropy and pseudo-orthogonality regularizations in the simulated multicomponent signal model. By drawing from concepts in statistics, the data-driven approach identifies global mcT_2_patterns in the WM, which are more likely to appear in the tissue, and are subsequently used to analyze the local signal in each voxel. This endows the resulting MWF values with high accuracy, precision, and robustness to noise when compared with the conventional RNNLS approach. The substantial difference in MWF values between healthy subjects and NAWM in people with MS indicates the potential of data-driven MWF values as a radiological biomarker for MS.

The ensuing values can be further extended to explore various aspects of the MS disease, including associated psychiatric conditions and cognitive impairment (Curti et al., 2018), optic neuritis (Reich et al., 2009), and remyelination (Caverzasi et al., 2023). The data-driven approach itself can be also utilized for a broader range of applications involving multicompartment analysis, such as fat/water separation (Nassar et al., 2023), analysis of the prostate (Storås et al., 2008), tumor characterization (Nikiforaki et al., 2020), and generalized to other types of contrasts.

## Data and Code Availability

The code used in this study will be shared publicly upon publication of the article.*In vivo*brain images presented in the study will be shared upon request, under approval of the local ethics committee and a data-transfer agreement.

## Author Contributions

S.Z.: Methodology, Software, Validation, Formal analysis, Investigation, Writing—Original draft, Visualization; N.O.: Software, Investigation; D.R.: Investigation, Resources; N.S.: Investigation; T.B.-K.: Resources, Project administration; D.B.-A.R.: Resources, Validation; S.S.: Resources, Validation; C.H.: Resources, Validation; N.B.-E.: Conceptualization, Methodology, Software, Writing—Review & Editing, Visualization, Supervision.

## Funding

This work was supported by Ichilov-TAU joint program for innovation in medical engineering (Alrov fund).

## Declaration of Competing Interest

The authors declare no competing interests.

## Supplementary Materials

Supplementary material for this article is available with the online version here:https://doi.org/10.1162/imag_a_00254.

## Supplementary Material

Supplementary Material

## Appendix A: Matlab’s Quadprog Solver

The minimization problem inEq. (12)was solved using Matlab’s quadprog solver (Mathworks, version 2022b) for quadratic problems with linear constraints:

$$\underset{x}{\text{argmin}}\frac{1}{2}x^{T}Hx+q^{T}x\;such\;thatx_{i}\geq 0$$
(A. 1),

where H is semipositive definite matrix and q is a linear term. The optimization problem is cast into the quadratic programming solver by rearrangingEq. (12):

$$\frac{1}{2}{\left\|DW-s\right\|}_{2}^{2}+\lambda_{Tik}{\left\|W\right\|}_{2}^{2}+\lambda_{L_{1}}\left\|W\right\|=$$
(A. 2)

$$=\frac{1}{2}{\left(DW-s\right)}^{T}\left(DW-s\right)+\lambda_{Tik}W^{T}W+\lambda_{L_{1}}\left\|W\right\|,$$

whereDis a dictionary of selected mcT_2_motifs,$W\;\in {\mathbb{R}}^{\left|D\right|}$is the unknown vector of weights of the elements inD,|D|is the number of elements inD, and$\lambda_{Tikh},\;\lambda_{L_{1}}\;\geq 0$are Tikhonov and L_1_regularization weights. To keep only physical solutions, we constrain the elements inWto be non-negative, i.e.,$W_{i}\geq 0$so$\left\|W_{1}\right\|=\sum_{i}\left|W_{i}\right|=\sum_{i}W_{i}$. Rearranging the last expression yields

$$\frac{1}{2}{\left(DW\;-s\right)}^{T}\left(DW\;-s\right)+\lambda_{Tik}W^{T}W\;+\lambda_{L_{1}}\left\|W\right\|=$$
(A. 3)

$$=\frac{1}{2}\left(W^{T}D^{T}DW\;-2s^{T}DW+s^{T}s\right)+\lambda_{Tik}W^{T}W\;+\lambda_{L_{1}}\sum_{i}W_{i}.$$

The term$s^{T}s$does not depend onWand thus has no effect on the minimization and can be discarded:

$$\frac{1}{2}W^{T}D^{T}DW-S^{T}DW+\lambda_{Tik}W^{T}W+\lambda_{L_{1}}\sum_{i}W_{i}=$$
(A. 4)

$$=\frac{1}{2}W^{T}\left(D^{T}D+2\lambda_{Tik}I\right)W-s^{T}DW+\lambda_{L_{1}}{\text{1}}^{T}W$$

$$=\frac{1}{2}W^{T}\left(D^{T}E+2\lambda_{Tik}I\right)W+\left(\lambda_{L_{1}}{\text{1}}^{T}\;-s^{T}D\right)W,$$

whereIis the identity matrix and${\text{1}}^{T}$is a vector of ones. Using the expression inEq. (A.1), we can calculate that$H\;=D^{T}D+2\lambda_{Tik}I$and$q^{T}\;=\lambda_{L_{1}}{\text{1}}^{T}\text{​}-S^{T}D$.
