# Supplementary material for: Improved myelin water imaging using B 1 + correction and data-driven global feature extraction: Application on people with MS
Source: Imaging Neurosci (Camb). 2024 Jul 31;2:imag-2-00254. doi: 10.1162/imag_a_00254 (PMC12272186; doi:10.1162/imag_a_00254)
Supplement: Supplementary Material [file imag_a_00254-supp.pdf]

Improved myelin water imaging using B_1^+ correction and data-driven global feature extraction: application on people with MS

SUPPLEMENTARY MATERIALS

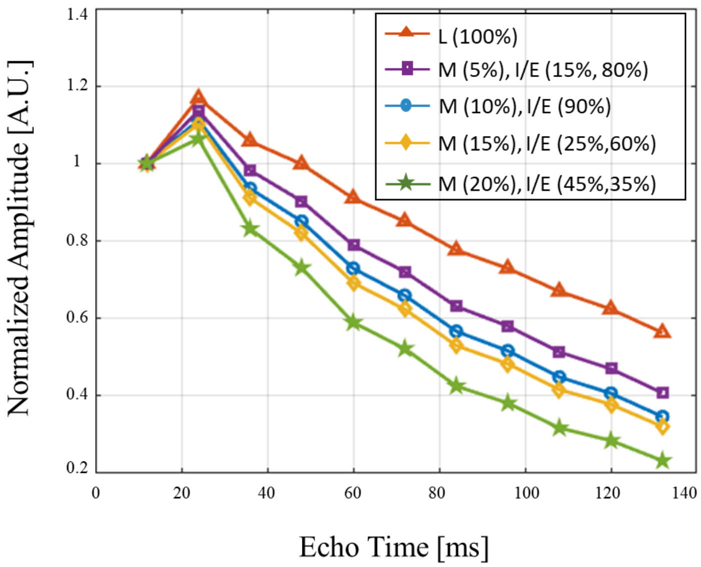

Tissue type	Relative fractions [%]	T_2 values [ms]
△	[100]	[120]
□	[5,15,80]	[30,70,90]
○	[10,90]	[10,75]
◇	[15,25,60]	[20,60,80]
★	[20,45,35]	[15,50,70]

Fig. S1: Representative simulated signals of the numerical phantom used in this study, providing ground truth reference values. All curves were simulated using B_1^+ = 100%. L-lesion, M-myelin, I/E-intra/extracellular.

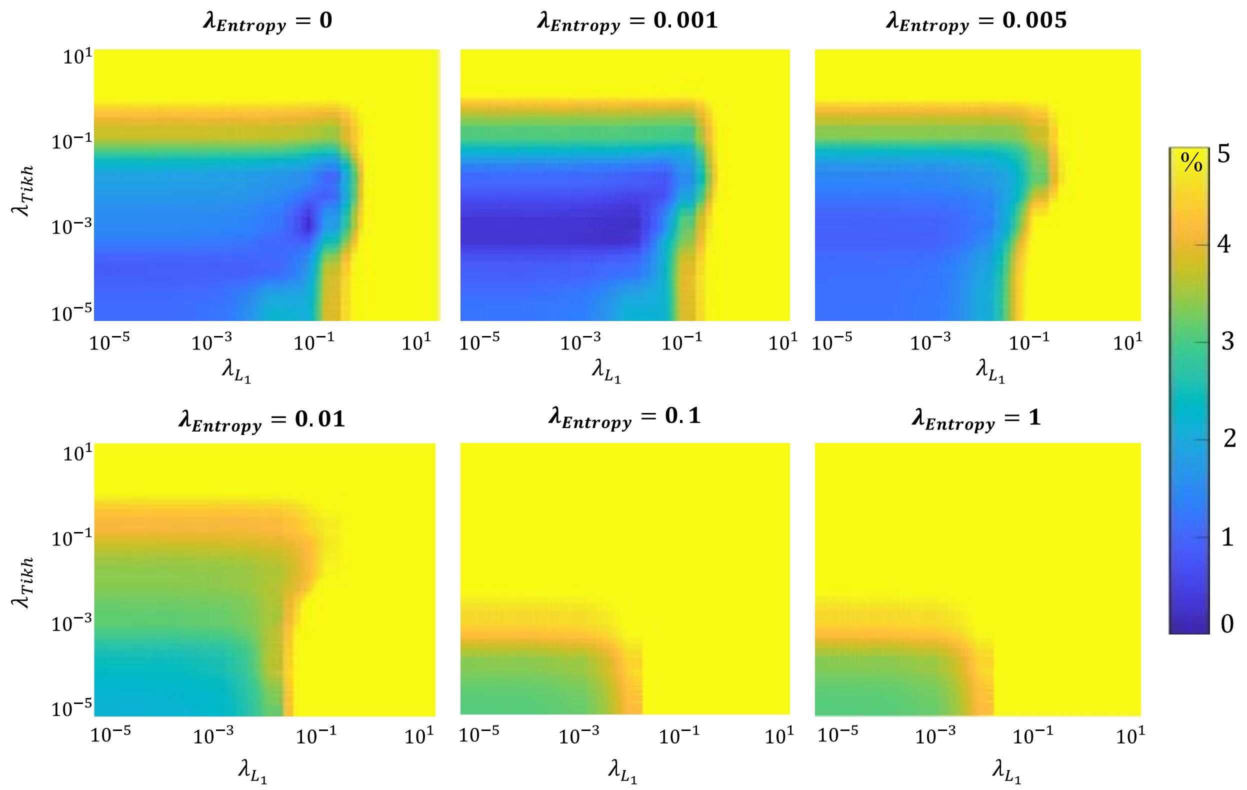

Fig. S2: Mean absolute error [%] of fitted MWF values as function of L_1 and Tikhonov regularization weights for numerical phantom at SNR=500. Six different entropy regularization values are shown between $\lambda_{Entropy} = 0$ (no regularization) to $\lambda_{Entropy} = 1$ (strong regularization).

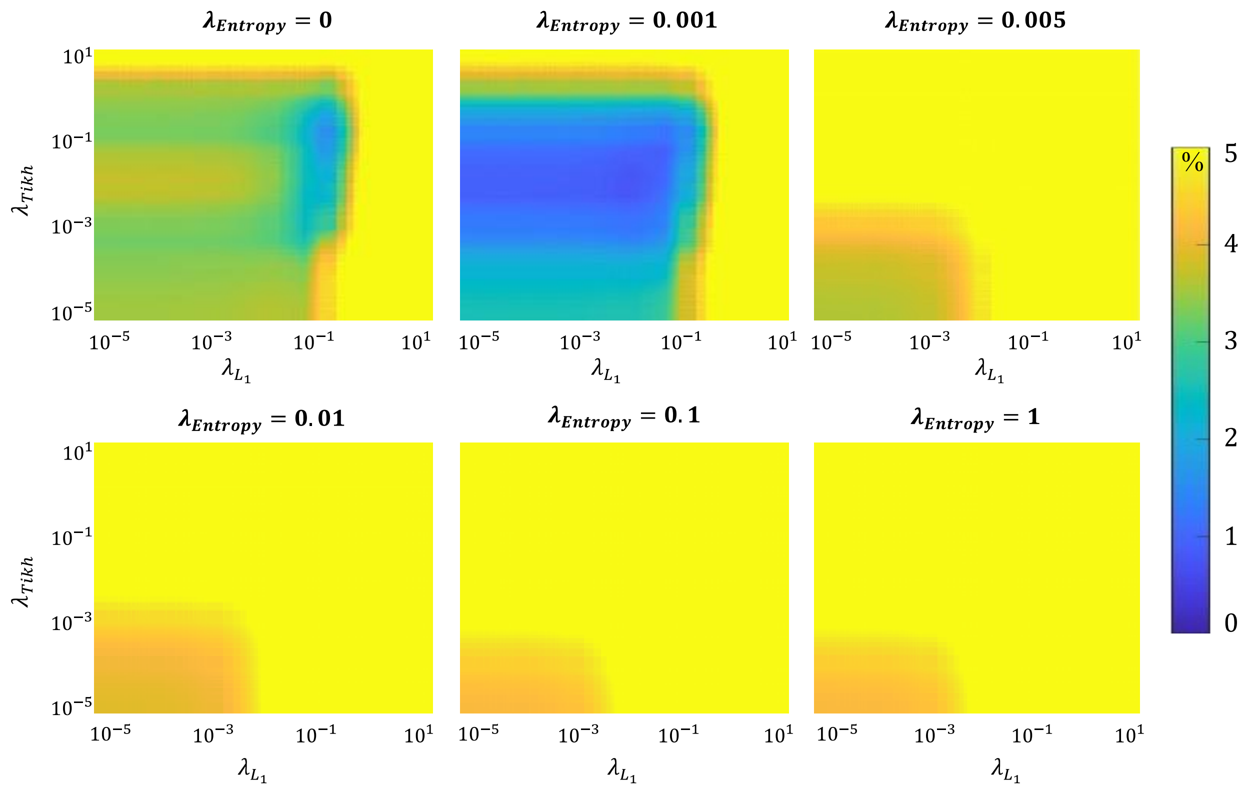

Fig. S3: Mean absolute error [%] of fitted MWF values as function of L_1 and Tikhonov regularization weights for numerical phantom at SNR=100. Six different entropy regularization values are shown between $\lambda_{Entropy} = 0$ (no regularization) to $\lambda_{Entropy} = 1$ (strong regularization).

Tube number	80 ms solution [%]	60 ms solution [%]	20 ms solution [%]
1	100.0	0.0	0.0
2	86.0	14.0	0.0
3	81.9	14.3	3.8
4	78.8	14.1	7.1
5	74.5	14.5	11.1
6	71.4	14.3	14.3
7	66.8	14.6	18.5
8	63.8	14.5	21.7
9	59.0	14.8	26.2

Table S1: Composition of the physical phantoms used in the study. Each "phase" of the phantom consisted of different combination of three types of tubes, containing $MnCl_2$ solutions with T_2 relaxation times of 80, 60, and 20 ms.

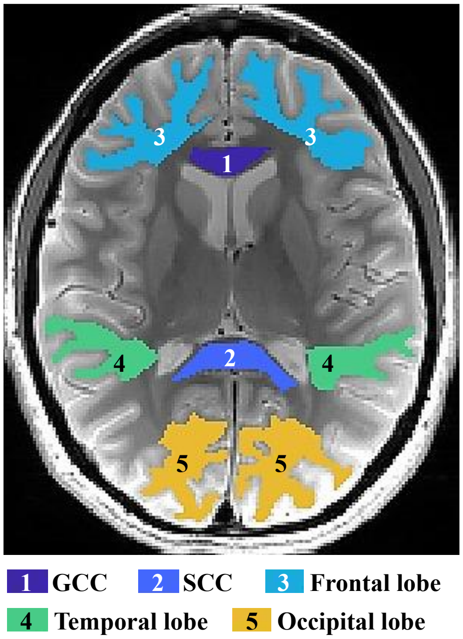

Fig. S4: An example of manually segmented ROI's, used in the statistical analysis shown in Table S2 and Table S3.

**MWF values [%]**	Healthy subjects	MS patients	P-value
GCC	13.6 ± 1.8	10.4 ± 2.3	<0.0001
SCC	15.2 ± 1.8	9.6 ± 1.8	<0.0001
Frontal Lobe	11.6 ± 1.8	9.2 ± 1.7	<0.0001
Occipital Lobe	13.1 ± 1.6	9.2 ± 1.6	<0.0001
Temporal Lobe	12.9 ± 1.7	9.0 ± 1.8	<0.0001
White Matter	13.0 ± 1.6	9.2 ± 1.7	<0.0001

Table S2: Mean and SD of MWF values [%] across 26 healthy subjects and 29 people with MS. Values are shown for six regions of interest in the NAWM, calculated using the proposed data-driven technique. Statistically significant results compared to the healthy subjects are shown using p-value.

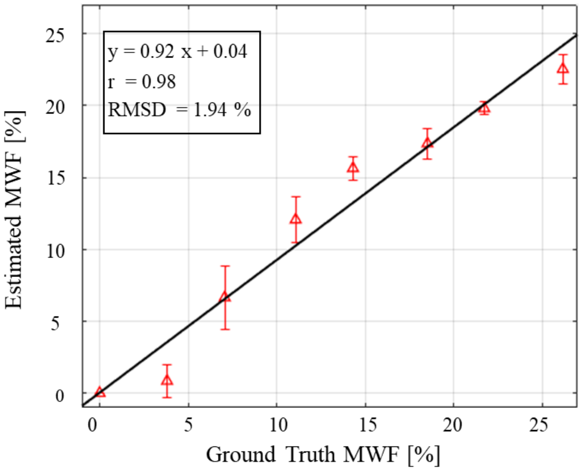

Fig. S5: MWF values for the physical phantom, computed using conventional mcT_2 fitting. Mean and SD (error bars) of MWF are shown for eight tubes, containing increasing levels of a fast-relaxing component having T_2 = 20 ms (see Table S1, and Fig. 4 in the main manuscript). Black line denotes the best fit line. Correlation value of r = 0.98 and absolute error of 1.56 ± 1.24% were achieved between the estimated and ground truth MWF values. Notably, these results were obtained after separately optimizing the reconstruction parameters to the conventional fitting.

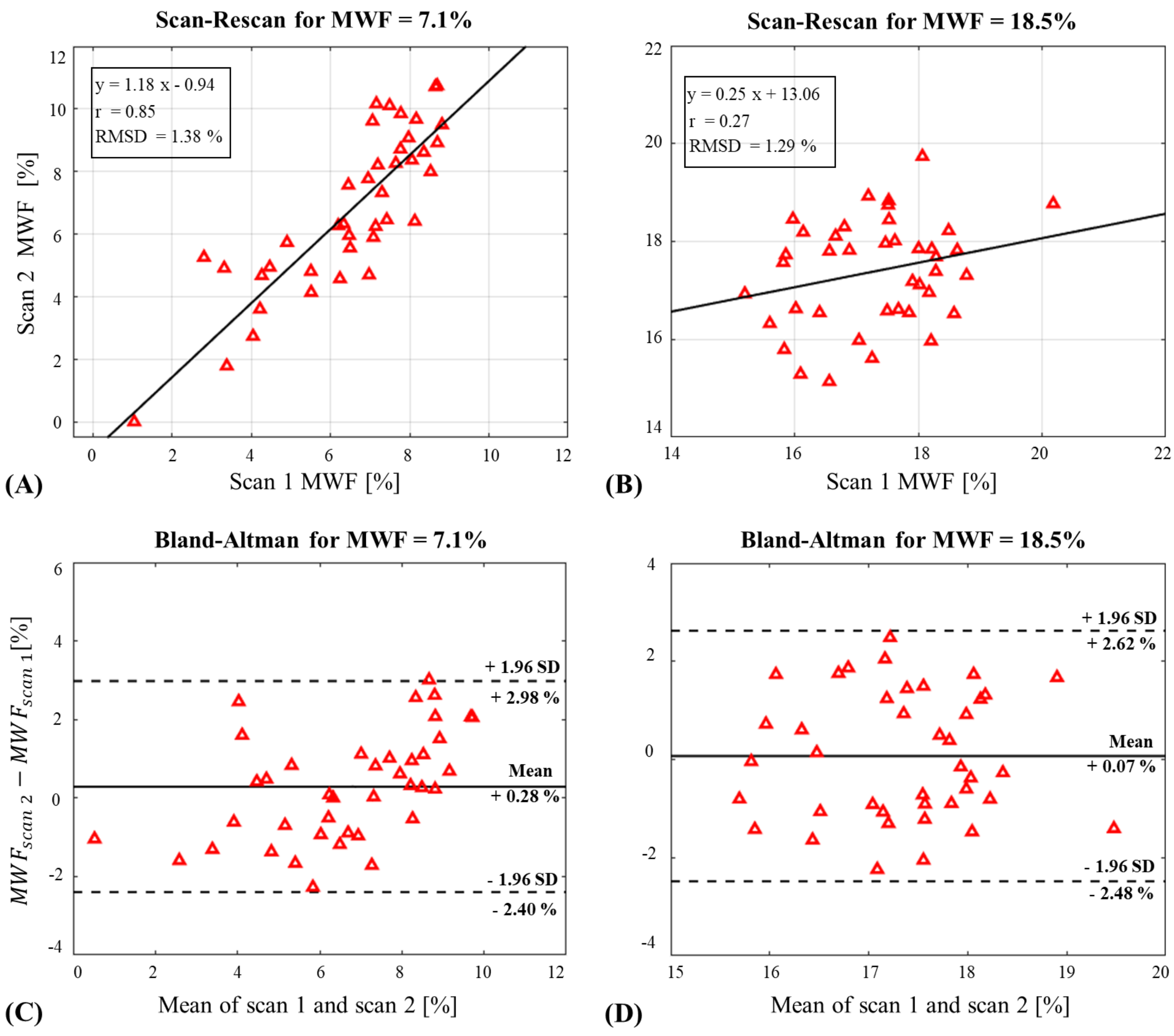

Fig. S6: Scan-rescan analysis and Bland-Altman plot for MWF values derived using the conventional approach for two tubes containing **(A,C)** 7.1% and **(B,D)** 18.5%. Spread of values is significantly larger compared to data-driven fitting (see Fig. 5), indicating larger sensitivity to random inter-scan signal variations and noise.

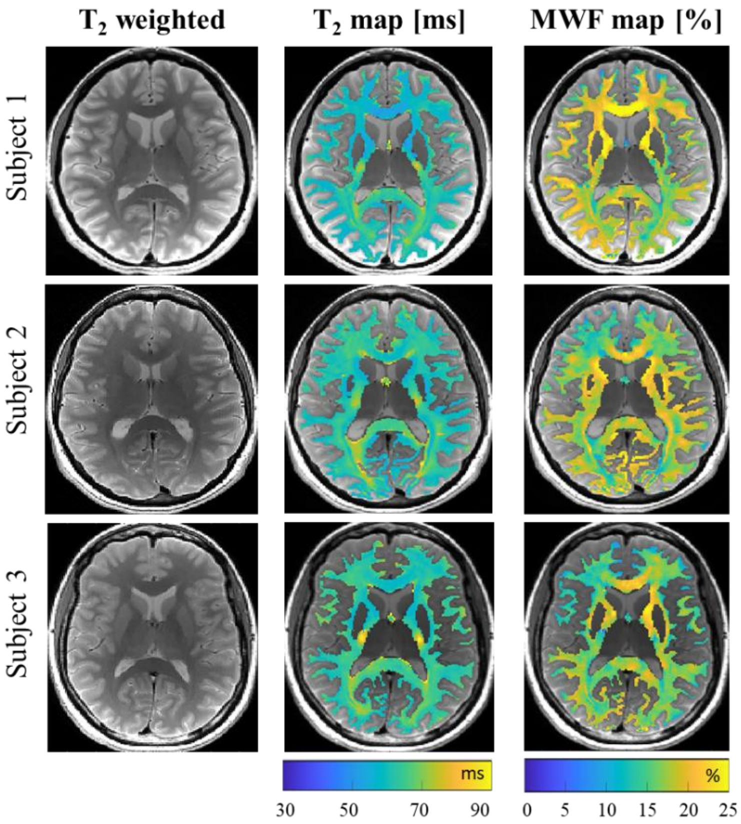

Fig. S7: T_2 weighted images, T_2 maps, and MWF maps for three healthy subjects, generated using conventional mcT_2 fitting. While T_2 maps are similar to ones obtained using data-driven fitting (see Fig. 6), MWF maps reflect higher variation and less physiological values.

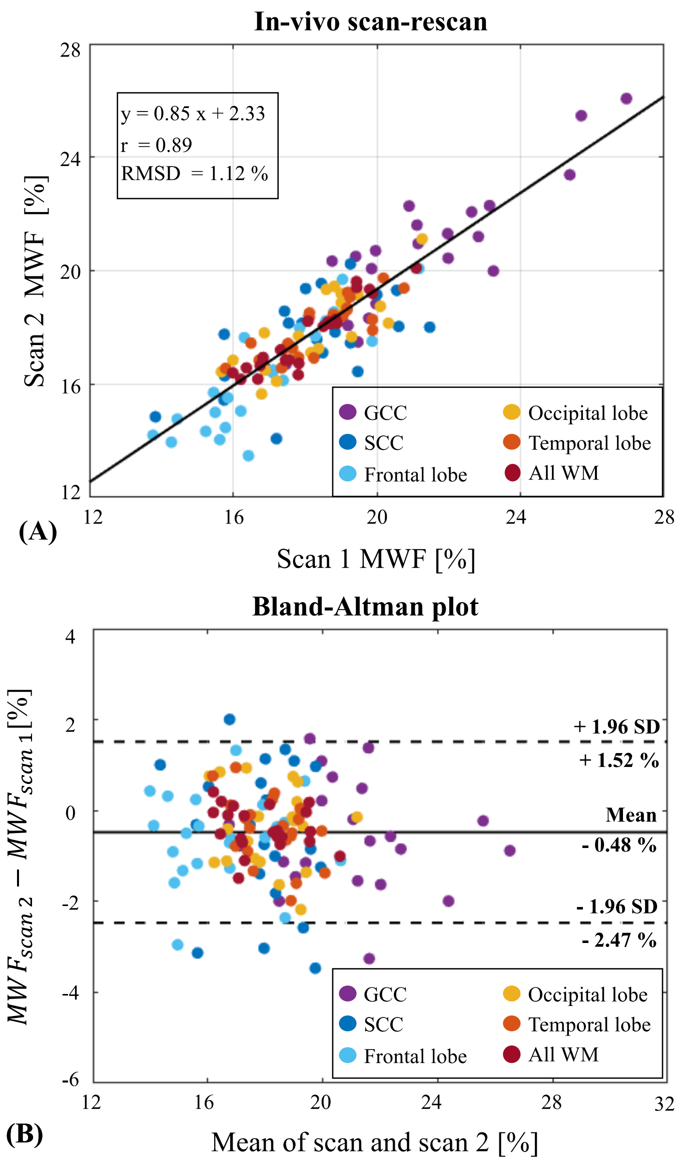

Fig. S8: (**A**) Scan-rescan analysis and (**B**) Bland-Altman plot for *In vivo* MWF values derived using the conventional approach.

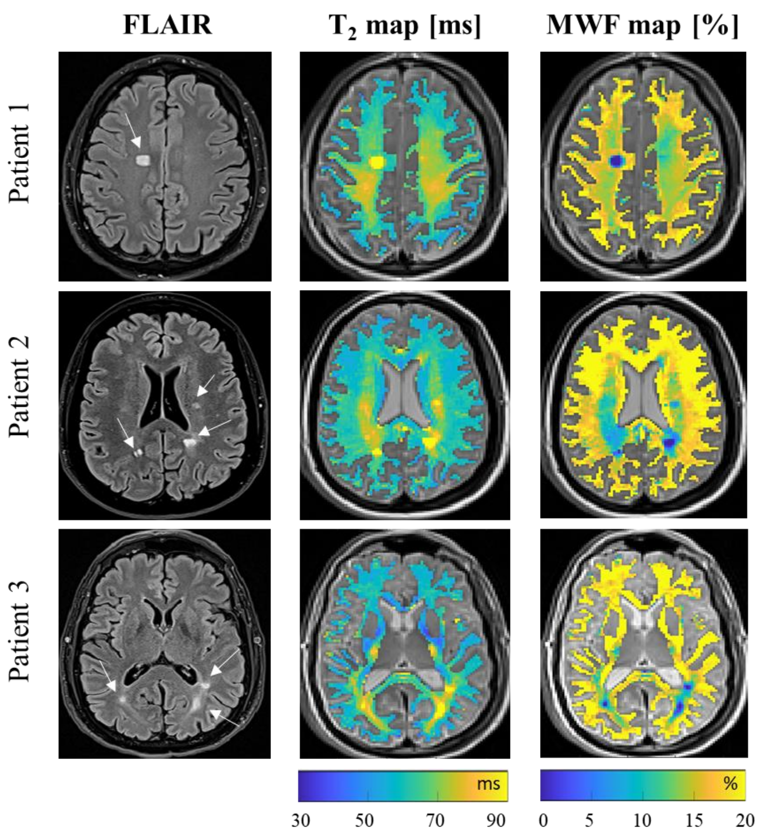

Fig. S9: FLAIR images, T_2 maps and MWF maps for three people with MS, generated using conventional mcT_2 fitting. While T_2 maps are similar to ones obtained using data-driven fitting (see Fig. 8), MWF maps reflect strong overestimation of myelin content, higher variability, and less physiological values.

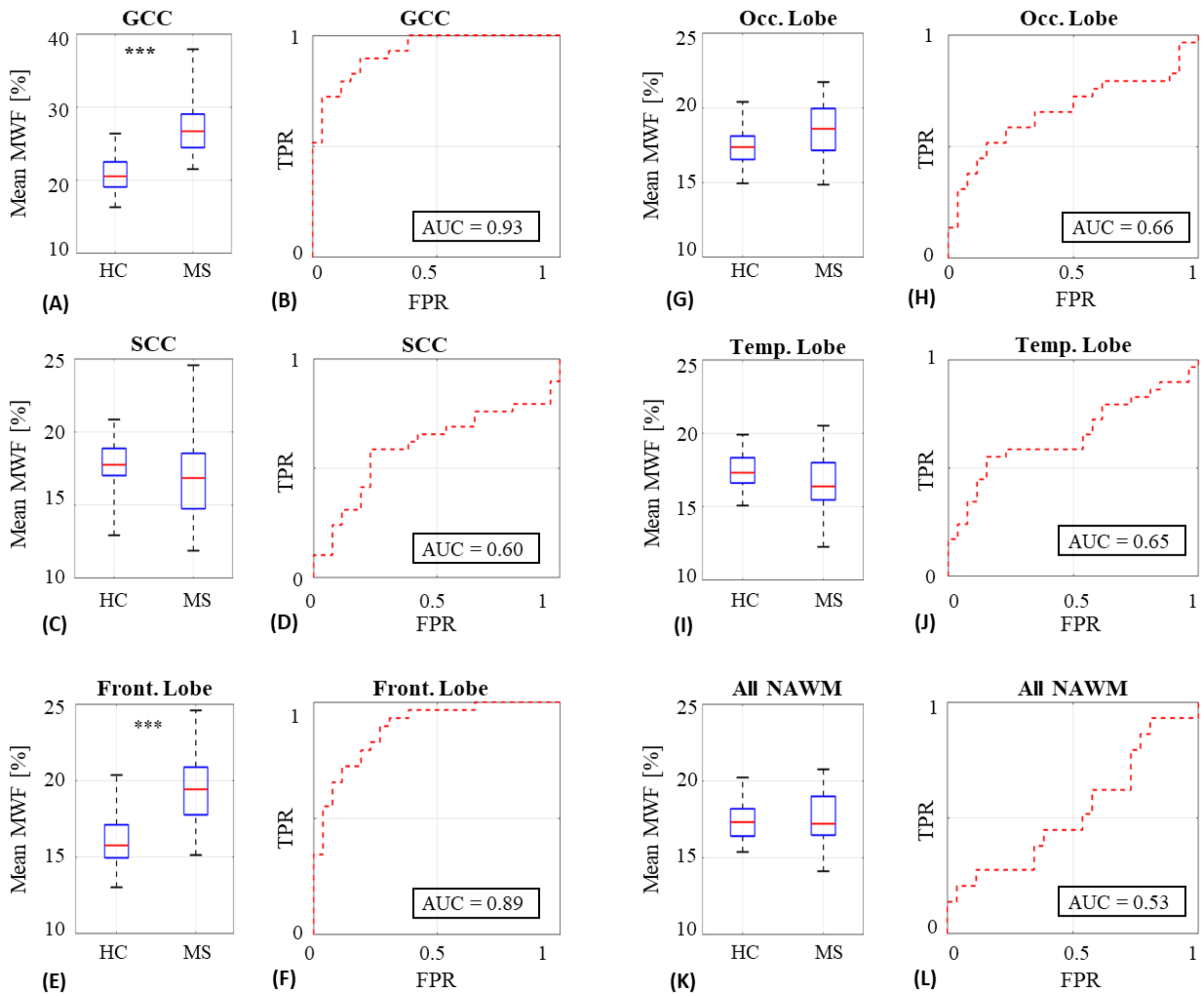

Fig. S10: Box plots of MWF values for six WM ROIs for healthy controls (**HC**) and people with MS, generated using conventional mcT_2 fitting. Statistically significant separation between the two groups was achieved for two ROIs (*** *p-value* < 0.0001, after correcting for multiple comparisons). ROC curves are shown on the 2nd and 4th columns, calculated based on mean MWF values in NAWM only (i.e., excluding lesions). **(A-B)** Genu of corpus callosum (GCC). **(C-D)** Splenium of corpus callosum (SCC). **(E-F)** Frontal (Front.) lobe. **(G-H)** Occipital (Occ.) lobe. **(I-J)** Temporal (Temp.) lobe. **(K-L)** All NAWM.

MWF values [%]	Healthy subjects	MS patients	P-value
GCC	20.9 ± 2.5	27.1 ± 3.6	<0.0001
SCC	17.5 ± 1.9	17.0 ± 3.0	0.4854
Frontal Lobe	16.1 ± 1.7	19.9 ± 2.3	<0.0001
Occipital Lobe	17.5 ± 1.3	18.4 ± 1.9	0.0418
Temporal Lobe	17.5 ± 1.2	16.6 ± 1.9	0.0583
White Matter	17.3 ± 1.2	17.6 ± 1.7	0.5434

Table S3: Mean and SD of MWF values [%] across 26 healthy subjects and 29 people with MS. Values are shown for six regions of interest in the NAWM, calculated using conventional analysis. Statistically significant difference (p-value < 0.05) was found for the frontal and occipital lobe. The GCC region also showed statistically significant difference, yet in the "opposite" direction, indicating higher myelin content in the group of people with MS.
